# Molecular Design and Experimental Study of Deep Eutectic Solvents Extraction of Polydatin from *Polygonum cuspidatum*

**DOI:** 10.1021/acsomega.6c01051

**Published:** 2026-04-13

**Authors:** Xu Cai, Yuanguo Xiong, Zhouwei Hong, Tong Xia, Dan Li, Liu Tang

**Affiliations:** † Department of Pharmacy, 117921 Renmin Hospital of Wuhan University, Wuhan, Hubei 430000, P.R. China; ‡ Department of Engineering Physics, Tsinghua University, Beijing 100084, P.R. China; § College of Pharmacy, 240515 Hubei University of Chinese Medicine, Wuhan, Hubei 430000, P.R. China

## Abstract

As the main constituent of *Polygonum cuspidatum*, polydatin (PLD) possesses a diverse array of pharmacological activities. However, its poor water solubility and stability pose substantial challenges to its efficient extraction and further development. Deep eutectic solvents (DESs) are highly suitable media tailored to the molecular properties of active ingredients as well as their extraction and developmental requirements. According to the thermodynamic analysis of COSMO-RS, betaine (Bet)–glycerol (Gly) was identified as the optimal DES. And the optimal extraction conditions were established as follows: 28:1 mL/g of liquid–material ratio, 54 min of extraction time, and 246 W of ultrasonic power. The PLD yield was 20.34 ± 0.55 mg/g, which was 2.1-fold higher than that of ethanol extraction. Furthermore, the underlying extraction mechanism is explored by SEM, FT-IR, ^1^H NMR, and molecular dynamics simulations. The primary driving force underlying the extraction of PLD via Bet–Gly interactions is hydrogen bonding. And the most negative interaction energy between PLD and Bet–Gly reached −994.72 kcal/mol, whereas the highest hydrogen bond abundance (36.05%) was observed between PLD and Gly. Moreover, the DES-based extract exhibited excellent antioxidant activity, noncytotoxicity, and superior stability, which offer compelling evidence for its safe and effective application in multiple biomedical domains. Hence, this study provided a robust theoretical foundation for advancing the rational design of DES and the sustainable application of excess plant resources.

## Graphical abstract

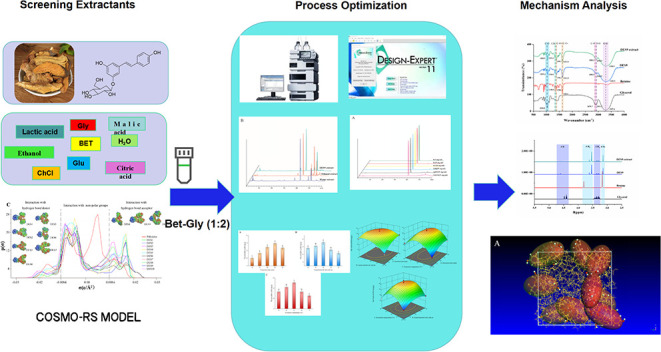

## 1 Introduction

*Polygonum cuspidatum* *Sieb. et Zucc.*, a traditional Chinese medicinal herb, is commonly utilized in the management of skin burns, infectious diseases, and jaundice.^1^ In Japan, the tender stems of tiger cane are a popular vegetable, celebrated for their crisp texture and distinctive sweet-tart flavor. However, the aggressive expansion of *P. cuspidatum* in Europe and North America has caused serious ecological harm, which is considered extremely hazardous to native plant communities.^2^ The development and utilization of its redundant resources have become an extremely pressing task. Polydatin (PLD) is the main active ingredient of *P. cuspidatum* and a key precursor for the production and preparation of resveratrol. It possessed a diverse array of pharmacological activities, including antioxidant, neuroprotective, antitumor, cardioprotective, and metabolic regulatory properties.^3^ Because of its superior antioxidant properties, PLD has become one of the most popular active additives in food and skin care products. Meanwhile, PLD has been developed as a nanocomplex for alleviating rheumatoid arthritis, a carboxymethyl chitosan delivery system against nephrotoxicity, and microneedles for treating arthritis.^4–6^ These innovations collectively provide a robust theoretical foundation for extensive applications of PLD. However, the water solubility and stability of PLD are unsatisfactory, which are significant challenges to the efficient extraction and development of PLD. Although PLD exhibits stronger antioxidant activity than resveratrol, its extraction from *P. cuspidatum* has been largely neglected.^7^ Deep eutectic solvents (DESs) are a highly suitable medium tailored to the molecular properties of active ingredients, as well as their extraction and developmental requirements. Hence, screening suitable DESs and exploring their application potential is of great significance for the sustainable application of excess plant resources.

Deep eutectic solvent (DES) is a new type of green, eco-friendly solvent composed of H-bond donors (HBD) and H-bond acceptors (HBA), which has attracted widespread attention in the pharmaceutical and chemical industries. Due to the plentiful intermolecular interactions, DES possessed distinctive merits such as remarkable solubilizing capacity, negligible volatility, and tunable molecular structures.^8^ And these exceptional physicochemical characteristics have rendered DES a widely utilized extraction medium; for instance, the extraction of resveratrol from *P. cuspidatum*, phenolic compounds from olive pomace, and phenolic compounds from *Moringa oleifera*.^9–11^ DES extracts demonstrated satisfactory extraction yields and exhibited notable antioxidant, antibacterial, and enzyme inhibitory properties. In addition, DESs are frequently utilized as powerful tools for strengthening the stability of active molecules. The retention rate of phenolic metabolites of *Moringa oleifera*, anthocyanins from *Perilla frutescens*, and flavonoids from jujubein DESs was superior to that stored in ethanol under various conditions.^11–13^ Hence, DESs have further consolidated their technical superiority in the extraction and storage of active ingredients, especially unstable molecules. Moreover, compared with conventional extraction methods, DES extraction avoids the application and contamination of organic solvents. And DES-based extracts are regularly employed as bioactive agents and modifiers in the production of packaging films, functional hydrogels, and cosmetics, which markedly simplify the complex separation process and product development.^14^ These results confirm the superiority and practicality of DES extraction.

DESs exhibit varying extraction capabilities in the specific extraction process for a variety of available HBDs and HBAs. However, screening suitable DESs through repetitive experiments is labor-intensive, time-consuming, and costly.^15^ Therefore, it is essential to employ reliable theoretical models for characterizing the thermodynamic properties of the research targets. The Conductor-like Screening Model for Real Solvents (COSMO-RS) represents a powerful computational tool for the theoretical prediction of thermodynamic characteristics and fluid phase equilibria of liquid mixture systems. It involves statistical thermodynamics and quantum chemistry as well as other theories from density functional theory. By inputting the relative structural information, it provides σ-profiles, solubility, and other information for research targets. This approach enables high-throughput prediction and screening of DESs for effectively extracting target substances.^15^ Under the guidance of relative solubility, choline chloride and acetamide were selected as the extraction solvents for polyphenolic compounds from *Camellia oleifera*.^16^ According to the activity coefficient at infinite dilution (AC^id^), glycerol and lactic acid were selected for enriching the essential oil of *Fructus Aurantii*.^17^ To recover lutein from *Scenedesmus sp*., a DES consisting of phenyl alcohol and thymol was chosen as the optimal DES.^18^ The predicted extraction trends of target compounds in different DESs closely align with the experimental outcomes. In comparison with the sophisticated experimental screening approach, COSMO-RS significantly reduces experimental workload as well as time consumption, while greatly improving the extraction efficiency of target compounds.^16–18^ Due to its robust thermodynamic calculation capabilities, COSMO-RS is a reliable and efficient tool for accurately predicting the optimal HBA and HBD combinations tailored to DES extraction systems.

Therefore, in this study, the optimum DESs for extracting the PLD from *P. cuspidatum* were investigated by integrating COSMO-RS and practical experiments. Subsequently, the ultrasound-assisted DES extraction (UA-DES) parameters for extracting the PLD form *P.cuspidatum* was optimized through single-factor experiments coupled with response surface methodology (RSM). Furthermore, the synergistic mechanistic effects of UA-DES on plant material were elucidated by integrating SEM, FT-IR, ^1^H NMR, and molecular dynamics simulation (MD). Finally, stability, antioxidant activity, and cytotoxicity experiments were conducted to identify the potential applications of DES-based extracts. This study not only establishes a robust theoretical foundation for the rational design and sustainable application of DESs in plant extraction but also furnishes novel insights into advancing green, efficient technologies for plant resource utilization.

## 2 Materials and Methods

### 2.1 Experimental Materials and Reagents

*Polygonum cupidatum* was sourced from Qianwengtang Pharmaceutical Co., Ltd. (Bozhou, China). After low-temperature drying, the moisture content was controlled to below 10%. Polydatin (PLD), betaine (Bet), choline chloride (ChCl), and ferrous sulfate were obtained from Zhuhai Lizhu Reagent Co., Ltd. (Zhuhai, China). Thiazolyl blue tetrazolium bromide (MTT), fetal bovine serum (FBS), trypsin, DMEM medium, and salicylic acid were purchased from Shanghai Yuanye Biotechnology Co., Ltd. (Shanghai, China). 1,1-Diphenyl-2-picrylhydrazine (DPPH), malic acid (MA), citric acid (CA), glucose (Glu), lactic acid (LA), glycerol (Gly), acetonitrile, ethanol, and hydrogen peroxide (H_2_O_2_) were obtained from Bosi Biotechnology Co., Ltd. (Wuhan, China).

### 2.2 Conductor-Like Screening Model for Real Solvent Simulations

The molecular structures of PLD, HBAs, and HBDs are illustrated in Table S1. PLD, Bet, and ChCl were designated as the solute and HBA, respectively. First, the geometric optimizations of PLD, HBAs, HBDs, and DESs were performed through TURBOMOLE software for obtaining the surface charge distribution parameters. The calculation parameters of density functional theory (DFT) were set at the BP86/def-TZVP level of theory. Then, the generated COSMO files were exported to the COSMOthermX software in the form of DOL_TZVP_18 parametrization. The σ-profile, σ-potential, and AC^id^ for PLD in DESs were calculated at 298.15 K. The categories of DESs are as depicted in Table S2.

### 2.3 Extraction Process of Polydatin and Experimental Verification

#### 2.3.1 Preparation of Deep Eutectic Solvents

All HBDs were individually mixed with BET and ChCl as depicted in Table S2, respectively. Subsequently, the resultant blends were transferred to a thermostatic water bath (Blend Hangzhou Baoheng Constant Temperature Technology Co., Ltd., Zhejiang, China) at 80 °C with continuous stirring, followed by the addition of 30 wt % water. After the powder had completely melted to form a homogeneous, transparent liquid, the resultant DESs were collected and stored in sealed vials for subsequent experiments.

#### 2.3.2 Extraction Process of Polydatin

The dried powder of *P. cuspidatum* was homogenized with DES at a liquid–material ratio of 10:1 mL/g and treated with a KQ-300DE ultrasonic cleaner at 250 W (Kun Shan Ultrasonic Instruments Co., Ltd., Kunshan, Jiangsu, China) at 25 °C for 60 min. The molar ratios and water contents of the suitable DES were set as specified in Table S3. Thereafter, the mixture was subjected to LG16-W high-speed centrifugation (Beijing Jingli Centrifuge Co., Ltd., Beijing, China) at 9000 rpm. After 10 min, the PLD concentration in DES was analyzed using the HPLC system, and the appropriate suitable DES was screened on the basis of the extraction efficiency.

### 2.4 Optimization of the Extraction Process

Single-factor experiments were conducted with the PLD yield designated as the key evaluation index, where the levels of each independent variable were set as specified in Table S3. Based on the experimental results of the single-factor analysis, a three-factor, three-level Box–Behnken design was implemented to optimize the PLD extraction process and analyze the interactive effects of variables. Design Expert 13 software was employed to develop the coding levels, and the experimental levels for RSM factors are presented in Table S4.

### 2.5 HPLC Analysis

The HPLC system conditions were derived from the established analysis method,^19^ with appropriate minor adjustments to accommodate the matrix characteristics of DES-based extracts. The sample solutions were analyzed by the LC-2030 Plus HPLC (Shimadzu Co., Kyoto, Japan) with a Shim-pack STR ODS-II column (4.6 × 250 mm, 5 μm, Shimadzu Co., Kyoto, Japan). The column temperature was maintained at 30 °C. The mobile phase consisted of acetonitrile (Phase A) and water (Phase B), with gradient elution. Gradient elution programs: 5% Phase A from 0 to 3 min; 5–10% Phase A from 3 to 20 min; 10–40% Phase A from 20 to 60 min. The injection volume, flow rate, and detection wavelength were set at 10 μL, 1.0 mL/min, and 310 nm, respectively.

A series of PLD reference substance solutions was prepared and analyzed. The mass concentration and peak area of the PLD solution were set as X_1_ (mg/mL) and Y_1_, respectively. The standard curve equation was carried out: Y_1_ = 51166991.7X_1_ – 251509.9 (R^2^ = 0.999). And the PLD yield (Y_2_, %) was calculated using eq 1.

1 $$Y_{2}=\frac{X_{1}\times V}{m}$$

where X_1_ represented the PLD concentration, mg/mL; V was the volume of DES solution, mL; and m referred to the dry weight of *P. cuspidatum* powder, g.

### 2.6 Characterization of Deep Eutectic Solvents

#### 2.6.1 Fourier Transform Infrared Spectroscopy Analysis

FT-IR spectroscopy was utilized as a spectroscopic tool to characterize the intermolecular interaction mechanisms among the constituent components within the DES extract. DES9, DES9 extract, Bet, and Gly were pressed into thin slices and placed on a KBr disc, and then analyzed by IR Spirit-X Fourier transform infrared spectroscopy (Shimadzu Co., Kyoto, Japan). The spectral acquisition range was precisely set to 4000–400 cm^–1^, and all spectral data were systematically recorded for subsequent peak assignment and interaction analysis.

#### 2.6.2 Hydrogen Nuclear Magnetic Resonance Analysis

^1^H NMR was utilized for detecting the interaction effects between the constituent components within the DES extract. DES9, DES9 extract, Bet, and Gly were dissolved in D_2_O and transferred to a Fourier 80 Desktop NMR spectrometer (Bruker Co., Billerica, MA, USA) at 25 °C. The corresponding chemical shift data were processed and analyzed by using MestReNova software.

#### 2.6.3 Scanning Electron Microscope Analysis

The extraction residues of *P. cuspidatum* obtained via UA-DES and DES9 extractions were rinsed thoroughly with deionized water three times and subjected to freeze-drying. Subsequently, these samples were gold-coated and mounted on aluminum stubs for further analysis. The SEM images of the original powder and DES9 and UA-DES extraction residues of *P. cuspidatum* were observed by the EVO 15 SEM (Carl Zeiss AG, Jena, Thuringia, Germany).

#### 2.6.4 Molecular Dynamic Simulation of Polydatin in DES

MD simulations of DES-PD were conducted using the Amorphous Cell (AC) module of 2023 Materials Studio (MS) software. Initially, the structural files of PLD, HBAs, and HBDs were downloaded from PubChem (https://pubchem.ncbi.nlm.nih.gov/), then performed by the DMol3 module with the GGA/BLYP functional. And ESP charges were assigned to the structures. After that, the structures of PLD, HBAs, and HBDs were optimized using the Forcite module with the COMPASSIII force field and a total of 5000 optimization cycles. For the construction of DES models, the optimized structural models were imported into the AC module. Based on the pre-experiments of PLD solubility, the cubic simulation boxes with periodic boundary conditions were configured as detailed in Table S3. After that, geometric optimization of the AC models was also performed by the Forcite module, followed by NVT and NPT ensemble MD simulations. A time step of 1 fs was adopted for both the NVT and NPT simulation stages with the total simulation duration set to 100 ps. Finally, the binding energy and H-bond were calculated and analyzed.

## 3 Results and Discussion

### 3.1 Thermodynamic Analysis of Polydatin Properties

The extraction efficiency depends on both the inherent characteristics of the solute–solvent system and the tunable external process parameters. Its magnitude is determined by the synergistic interplay of these multiple factors. The solubility parameter played a crucial role in the extraction of the target compound.^20^ A comprehensive thermodynamic analysis of the solutes was helpful for the design of DESs. COSMO-RS was an effective predictive tool, capable of deriving the thermodynamic characteristics of the target molecule from theoretical data.^21^ This capability facilitated the elucidation of solubility tendencies, including the σ-profile, σ-potential, and AC^id^. The σ-profile quantitatively described the local polarity distribution surface properties of target molecules and could be categorized as HBD, nonpolar, and HBA regions.^22^ These three regions are specifically defined: the negative charge attraction region (HBD), the nonpolar region, and the positive charge attraction region (HBA) are characterized by σ < −0.0082 e/Å^2^, −0.0082 < σ < 0.0082 e/Å^2^, and σ > 0.0082 e/Å^2^, respectively. The visualization revealed the molecular conformation and surface charge distribution, where red indicated negative charges, blue represented positive charges, and green signified neutral charges.

PLD, the main active component of *P. cuspidatum*, exhibited antioxidant, neuroprotective, antitumor, cardioprotective, and metabolic regulatory properties.^23^ As illustrated in Figure 1A, the majority of the σ-profile of PLD was localized within the nonpolar region, a key feature for predicting intermolecular interactions via COSMO-RS. This molecular feature was closely associated with the presence of benzene rings and the vinyl group in PLD. Hence, PLD was a hydrophobic molecule. Meanwhile, the green regions on the electrostatic potential diagram confirmed the hydrophobicity of PLD. PLD exhibited distinct peaks in the polar regions corresponding to HBA and HBD regions. These characteristics revealed that PLD had dual HBA and HBD properties. Notably, the peak width in the HBD region was wider than that in the HBA region, suggesting the predominance of the HBD characteristics in PLD.

**Figure 1:**
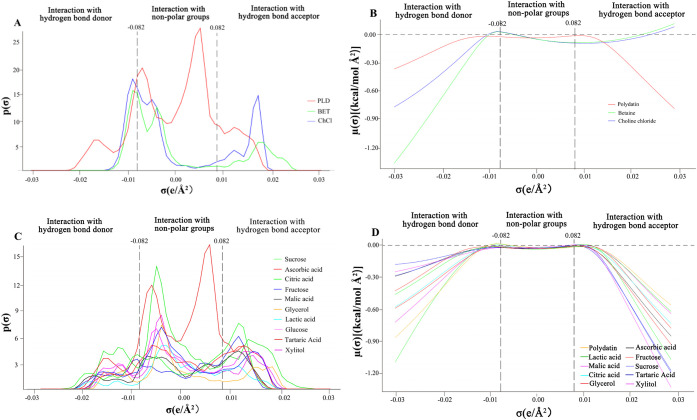
σ-Profile and σ-potential diagram. **A** The σ-profile diagram of PLD and HBAs, **B** the σ-potential diagram of PLD and HBAs, **C** the σ-profile diagram of HBDs, and **D** the σ-potential diagram of HBDs.

The σ-potential refers to a thermodynamic parameter that quantitatively describes the intermolecular affinity between different components. A more negative μ­(σ) value corresponded to stronger intermolecular attractive interactions, whereas a more positive value was indicative of enhanced intermolecular repulsive forces. Similar to the σ-profile, the σ-potential was also categorized into three distinct regions, namely the HBD, nonpolar, and HBA regions.^24^ As presented in Figure 1B, the notably more negative σ-potential of PLD in the HBA region enhanced engagement with HBA. This strengthened contact originated mainly from electrostatic attraction between the electron-rich moieties of PLD and the electron-deficient sites of HBA molecules, thus enabling tighter molecular packing. Consequently, PLD exhibited higher solubility in DES that possessed a strong HBA character and hydrophobic groups.

### 3.2 Thermodynamic Analysis of DES Properties

As a novel category of eco-friendly solvents, DES was generated through the H-bond interactions between HBA and HBD. The primary physicochemical properties of DESs, including solubility, density, and viscosity, were predominantly determined by the intrinsic nature and interaction modes of the HBAs and HBDs.^25^ Hence, selecting an optimal combination of HBA and HBD was imperative to enhancing the yield of the target compound. As illustrated in Figure 1A, the σ-profile diagrams of Bet and ChCl exhibited distinct differences in both HBA and HBD polarity regions. Specifically, Bet displayed a broader peak width than ChCl in the HBA regions, whereas its peak width in the HBD regions was narrower than that of ChCl. The σ-potential diagram of HBAs in Figure 1B illustrates that Bet had a more negative value than ChCl in the HBD region. Hence, Bet was predicted to be the optimal HBA for interacting with PLD. The σ-profile diagrams of all HBDs are displayed in Figure 1C, and the distinct peaks in the polar regions of all HBDs are listed in Table S6. Among them, the peak width of Gly in the HBA region was wider than that in the HBD polarity region. In comparison to other candidate HBDs, the HBA regions of Gly exhibited exceptional performance. The results indicated that Gly had relatively stronger HBA characteristics.

The σ-profile diagrams of DES1–DES20 with the mole ratio 2:1 are displayed in Figure S1. And the distinct peaks in the polar regions of DES1–DES20 are presented in Table S7. As evidenced by the σ-profile results, all DESs displayed prominent peaks in the nonpolar region. Nevertheless, the nonpolar segments of DESs showed poor compatibility with PLD. Therefore, van der Waals forces played a secondary role in molecular interactions, while exerting a modest promoting effect on PLD extraction. The σ-potentials of candidate DESs were illustrated in Figure S1. All of the DESs exhibited an obvious negative value in the HBD region of σ-potentials, with DES9 having the second-highest absolute value. Conversely, DES9 displayed the minimum absolute value in the HBA region. These results demonstrated that DES9 possessed stronger HBA features and was capable of augmenting the combination with PLD in comparison with other DESs. However, it was challenging to screen large-scale solvents through theoretical analysis of the σ-profiles and σ-potentials.

### 3.3 The Activity Coefficient Prediction and Experimental Validation

As a critical fundamental parameter in solution chemistry, AC^id^ was primarily responsible for characterizing the inherent correlation between solute activity and concentration at low-concentration solutions. It reflected the nonideal behavior of solutes in dilute solutions, rendering it highly valuable for examining the thermodynamic characteristics of solutions.^26^ The AC^id^ of PLD in various DESs was simulated under DES1–DES20 (with a molar ratio of 2:1) at 25 °C. As depicted in Figure 2A, the order of the top DESs was as follows: DES9 < DES6 < DES4 < DES1 < DES20 < DES15 < DES16 < DES17 < DES12 < DES13. The extraction experiments of the top 10 DESs were also conducted. As depicted in Figure 2B, the extraction trends of all tested DESs were consistent with the predictions generated by COSMO-RS. DES9 achieved the highest yield of 17.44 ± 1.01 mg/g and was confirmed as the optimal DES for extracting PLD. Therefore, COSMO-RS was a more precise and rapid large-scale screening strategy for DES extraction. However, owing to its low viscosity and strong penetration ability, ethanol achieved a higher extraction rate than certain DESs. This phenomenon suggested that an extract solvent with a low viscosity coefficient can effectively reduce molecular diffusion resistanc, and enhance the overall extraction efficiency.^27^ Although the predictive accuracy of COSMO-RS was influenced by numerous dynamic factors, it remained feasible for screening solvent systems with comparable physicochemical properties.

**Figure 2:**
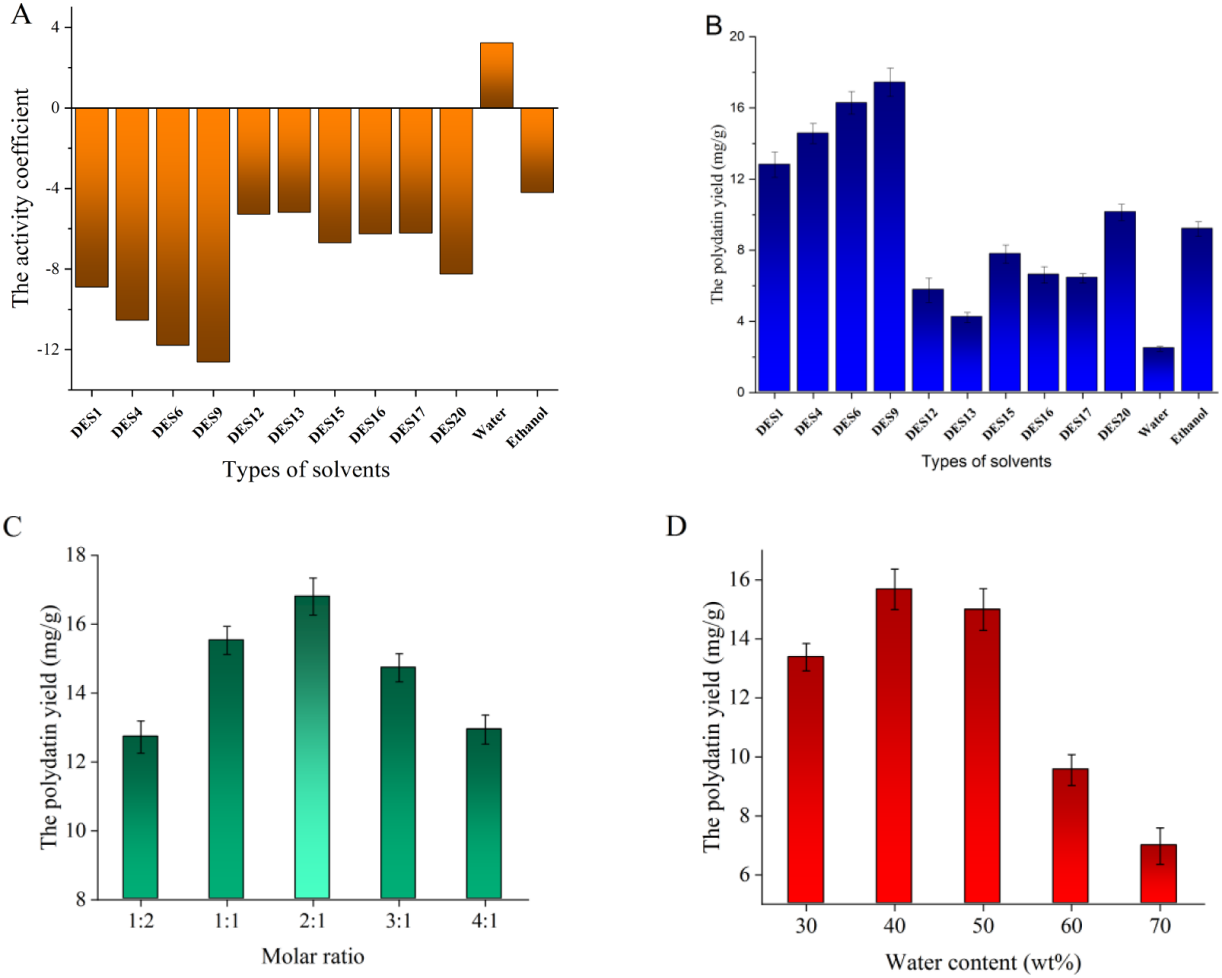
Predicted results of COSMO-RS and validation experiments. **A** The activity coefficients of PLD in DESs, **B** the yield of PLD extracted by the top 10 DESs, **C** the yield of PLD extracted by DES9 with various molar ratios, and **D** the yield of PLD extracted by DES9 with diverse water contents.

The rational regulation of molar ratio and water content was a necessary prerequisite for preparing DESs with excellent physicochemical properties.^28^ As illustrated in Figure 2C, the PLD yields of DES9 exhibited a trend of first increasing and then decreasing as the molar ratio increased. And the molar ratio of 2:1 for DES9 achieved the highest yield of 16.81 ± 0.54 mg/g. When the molar ratio of DES9 was less than 2:1, an increase in the molar ratio was beneficial for enhancing the H-bonding force of DES9, thus accelerating the dissolution of PLD. However, a molar ratio exceeding 2:1 impeded the dissolution kinetics of PLD because the dissolution efficiency of glycosides, pectin, and cellulose in DES9 declined noticeably.

Water acted as a crucial bridging agent in DESs, and the viscosity of DESs exhibited a nonlinear variation trend with increasing water content.^28^ As illustrated in Figure 2D, the PLD yield initially increased and then declined significantly with the increasing water content. Specifically, DES9 with 30 wt % water achieved the highest yield of 15.67 ± 0.69 mg/g. Below 40 wt % water content, increasing water addition helped reduce the viscosity of DES9 and enhance the mass transfer efficiency during PLD extraction. Once the water content exceeded 40 wt %, the viscosity and density change of DES9 on the PLD extraction could be ignored. However, the H-bonding force of DES9 was weakened. This was an unfavorable change for the dissolution of polysaccharides, pectin, and cellulose, which obstructed the PLD extraction.^29^ Consequently, the optimal DES was determined to be DES9 with a 2:1 molar ratio and 40 wt % water.

### 3.4 Optimization of the Polydatin Extraction Process

#### 3.4.1 Single-Factor Experiments

Numerous bioactive compounds have been efficiently extracted by employing diverse DESs. Multiple factors affected DES extraction results, with strong correlations to process parameters.^30,31^ Extraction time is a crucial factor governing the rate and efficiency of heat and mass transfer processes, as well as a fundamental guarantee for the sufficient diffusion of target compounds into DES.^30^ As exhibited in Figure S2-A, the PLD yield displayed a positive correlation with the increase in extraction time from 10 to 60 min, but further extension from 60 to 80 min led to a significant decrease in yield. In the solution extraction system, there was a distinct positive correlation between the molecular migration rate and the concentration gradient. It was primarily derived from the liquid–material ratio, which aligns with the fundamental principles of Fick’s diffusion law.^30^ As displayed in Figure S2-B, the PLD yield showed a positive correlation with the rising liquid–material ratio from 10:1 to 30:1 mL/g, whereas a marked reduction in yield occurred when the ratio was further elevated to 30:1 to 50:1 mL/g. UA offered a dependable strategy to counteract the adverse characteristics of DESs and plant substrates, achieved by transiently disrupting the rigid H-bonding networks of DESs and dense structural architectures of plant cells.^31^ As elucidated in Figure S2-C, the PLD yield exhibited a positive correlation with the increase in ultrasonic power ranging from 100 to 250 W. Conversely, the PLD yield decreased with the increase in ultrasonic power from 250 to 300 W. Extraction temperature was closely affiliated with the irregular thermal motion of molecules and also exerted a marked effect on the behavioral characteristics of DESs.^31^ And Figure S2-D showed that the PLD yield increased insignificantly with extraction temperature from 30 to 50 °C. Beyond 50 °C, it decreased as the extraction temperature increased. The variation trends of the experimental factors were consistent with those reported in prior studies, where DESs were applied for the extraction of essential oil from *Fructus aurantii*, polyphenols from *Cabernet sauvignon* seed, and ergosterol from *Lentinus edodes*.^17,29,32^ Consequently, extraction time, liquid–material ratio, and ultrasonic power were identified as the optimal process variables to be adopted in the subsequent experimental investigations.

#### 3.4.2 Response Surface Optimization Experiments

RSM was a statistical and mathematical method that involved fitting a polynomial model to data. Its corresponding analysis intuitively illustrated the regulatory roles of key variables in PLD yield, and clarified the factor hierarchy as well as optimal combination conditions.^33^ As summarized in Table 1, RSM was employed to design and optimize the levels of each factor. The regression equation of the PLD yield (Y, mg/g) was presented as eq 2:

2 $$Y=20.35-0.8432A-0.9223B-0.5933C-0.7476\mathrm{AB}-0.4207\mathrm{AC}+{0.1576\mathrm{BC}-A}^{2}-1.55B^{2}-3.28C^{2}$$

**Table 1:** Experimental Design by Box–Behnken and the PLD Yield

Code	Extraction time (A, min)	Liquid–material ratio (B, mL/g)	Ultrasonic power (C, W)	Yield of PLD (mg/g)
1	40	20:1	250	19.14
2	80	20:1	250	18.35
3	40	40:1	250	18.73
4	80	40:1	250	14.95
5	40	30:1	200	17.01
6	80	30:1	200	16.77
7	40	30:1	300	16.19
8	80	30:1	300	14.27
9	60	20:1	200	16.92
10	60	40:1	200	14.82
11	60	20:1	300	15.90
12	60	40:1	300	14.43
13	60	30:1	250	20.06
14	60	30:1	250	20.17
15	60	30:1	250	19.42
16	60	30:1	250	20.68
17	60	30:1	250	21.37

The results of the evaluation and analysis concerning the impacts of various factors on the PLD extraction are summarized in Table 2. The model *F* = 19.26, and *p* < 0.01, suggested that the model had good fitting reliability for the response variable. The regression model satisfactorily fitted the experimental data, with a nonsignificant lack-of-fit term (*F* = 0.745, *p* > 0.05), confirming no statistically significant misfit. From the model R^2^ = 0.9612 and R^2^_Adj_ = 0.9113, it could be inferred that the actual yield of PLD closely matched the predicted value. The hierarchical order of factors governing PLD extraction yield was established via *F*-value assessment, with the sequence being B > A > C. Steeper response surfaces and denser contour plot layouts enabled a clearer demonstration of the stronger interaction effects among the experimental factors.^33^ It can be observed from Figure 3A–F that the hierarchical order of factor interaction effects was ranked as BC < AC < AB. The optimal extraction process of PLD was identified as follows: liquid–material ratio 28:1 mL/g, extraction time 54 min, and ultrasonic power 246 W, with the theoretical yield of PLD being 20.61 mg/g. The actual yield of PLD was 20.34 ± 0.55 mg/g, which was 2.1-fold higher than that of ethanol extraction (9.72 ± 0.3 mg/mL). The results verified that DES9 was an efficient solvent for extracting PLD.

**Table 2:** Significance Test Report of Regression Model Coefficients on the PLD Yield

Parameter	Sum of Squares	Degree of Freedom	Mean Square	*F*-value	*p*-value
Model	82.93	9	9.21	19.26	0.0004^a^
A	5.69	1	5.69	11.89	0.0107^b^
B	6.81	1	6.81	14.22	0.007^b^
C	2.82	1	2.82	5.88	0.0457^b^
AB	2.24	1	2.24	4.67	0.0675
AC	0.708	1	0.708	1.48	0.2632
BC	0.0993	1	0.0993	0.2075	0.6625
A^2^	4.24	1	4.24	8.85	0.0207^b^
B^2^	10.08	1	10.08	21.07	0.0025^b^
C^2^	45.24	1	45.24	94.55	<0.0001^a^
Residual	3.35	7	0.4785		
Lack of Fit	1.2	3	0.4003	0.745	0.5788
Pure Error	2.15	4	0.5372		
Cor Total	86.28	16			

a Represents a highly significant difference (*p* < 0.01).

b Standsfor a significant difference (*p* < 0.05).

**Figure 3:**
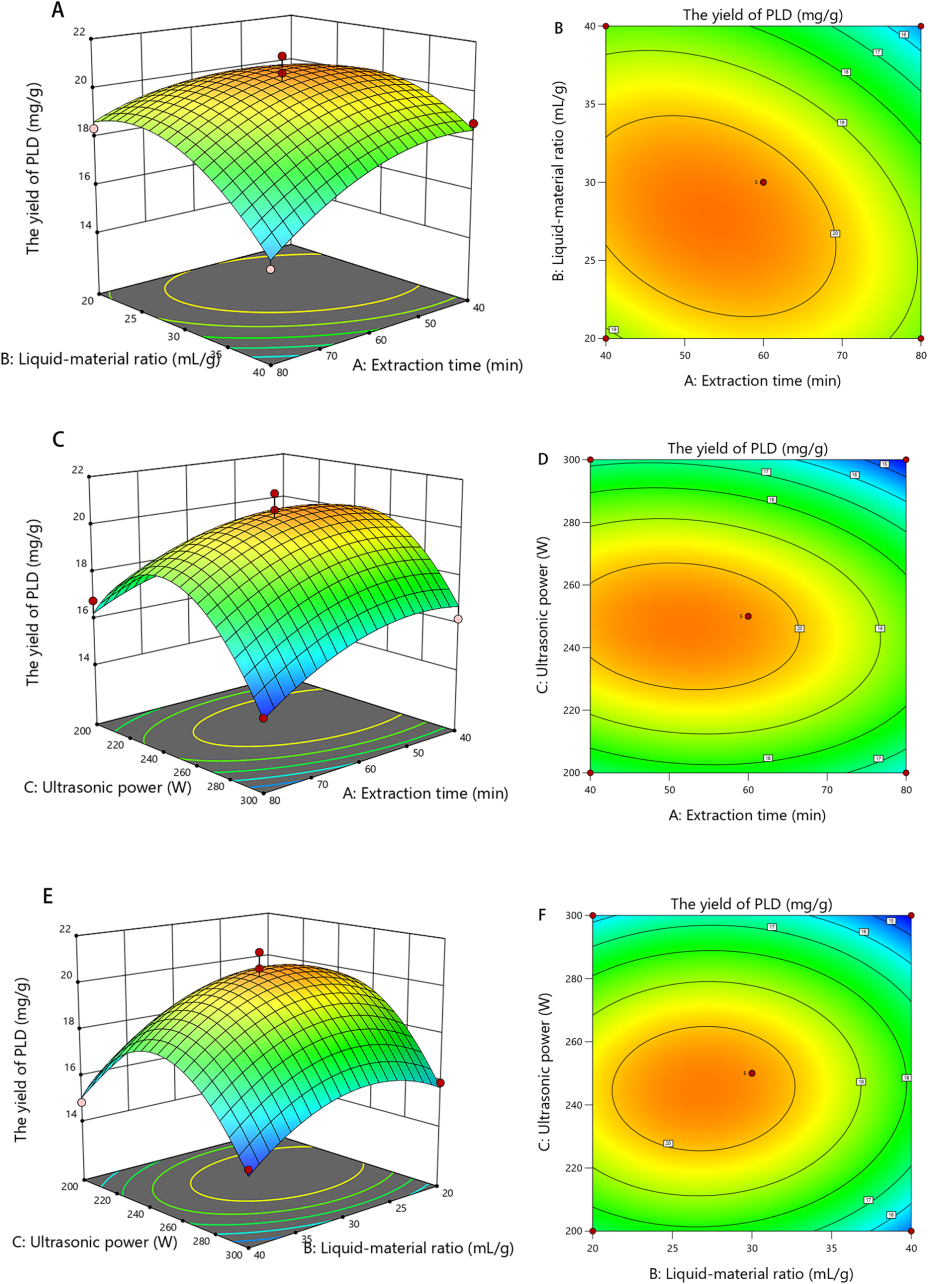
Response surface graph and contour plot reflect the interactive effects of extraction parameters on the PLD yield. **A, B** extraction time and liquid–material ratio; **C, D** extraction time and ultrasonic power; and **E, F** liquid–material ratio and ultrasonic power.

### 3.5 Characterization of Deep Eutectic Solvents

#### 3.5.1 Scanning Electron Microscope Analysis

Lignin, polysaccharides, and cellulose collectively constitute the rigid mechanical structure of plant cell walls, which act as the most formidable obstacles limiting the efficiency of plant extraction processes. SEM was an extremely versatile analytical instrument for examining the surface and cross-sectional morphology of samples.^34^ As illustrated in Figure 4A–B, the original powder of *P. cuspidatum* had a smooth and compact surface, which indicated the integrity of plant cells. The microstructures of *P. cuspidatum* powder treated by DES9 and UA-DES9 are depicted in Figure 4C–F, respectively. Compared with the original plant powders, a notable increase in surface pores and cracks was observed in the DES9 extraction group. This phenomenon could be attributed to the capacity of DES9 to impair the structural integrity of plant cell walls by degrading polysaccharides and cellulose, thereby promoting PLD release. Moreover, the powder of the UA-DES9 extraction group exhibited a considerably rougher surface than that of the standalone DES9 extraction group. This roughened surface was characterized by a dense array of concave–convex microstructures that bore a striking resemblance to corrosion-induced morphological features. Therefore, ultrasound could induce substantial damage to the cell wall structures via the cavitation effect, leading to more fragmented and disrupted architectural features. The PLD yield exhibited an initial increase with ultrasonic power from 100 to 250 W. However, the PLD yield decreased as the power was further elevated to 300 W. Excessive ultrasonic power reduced the PLD yield, which may be attributed to the structural damage or degradation of PLD induced by intense ultrasound.^29^ Consequently, SEM has indirectly demonstrated the excellent solubilizing effect of UA-DES9 on plant cell walls, which constitutes a crucial factor for improving the PLD yield

**Figure 4:**
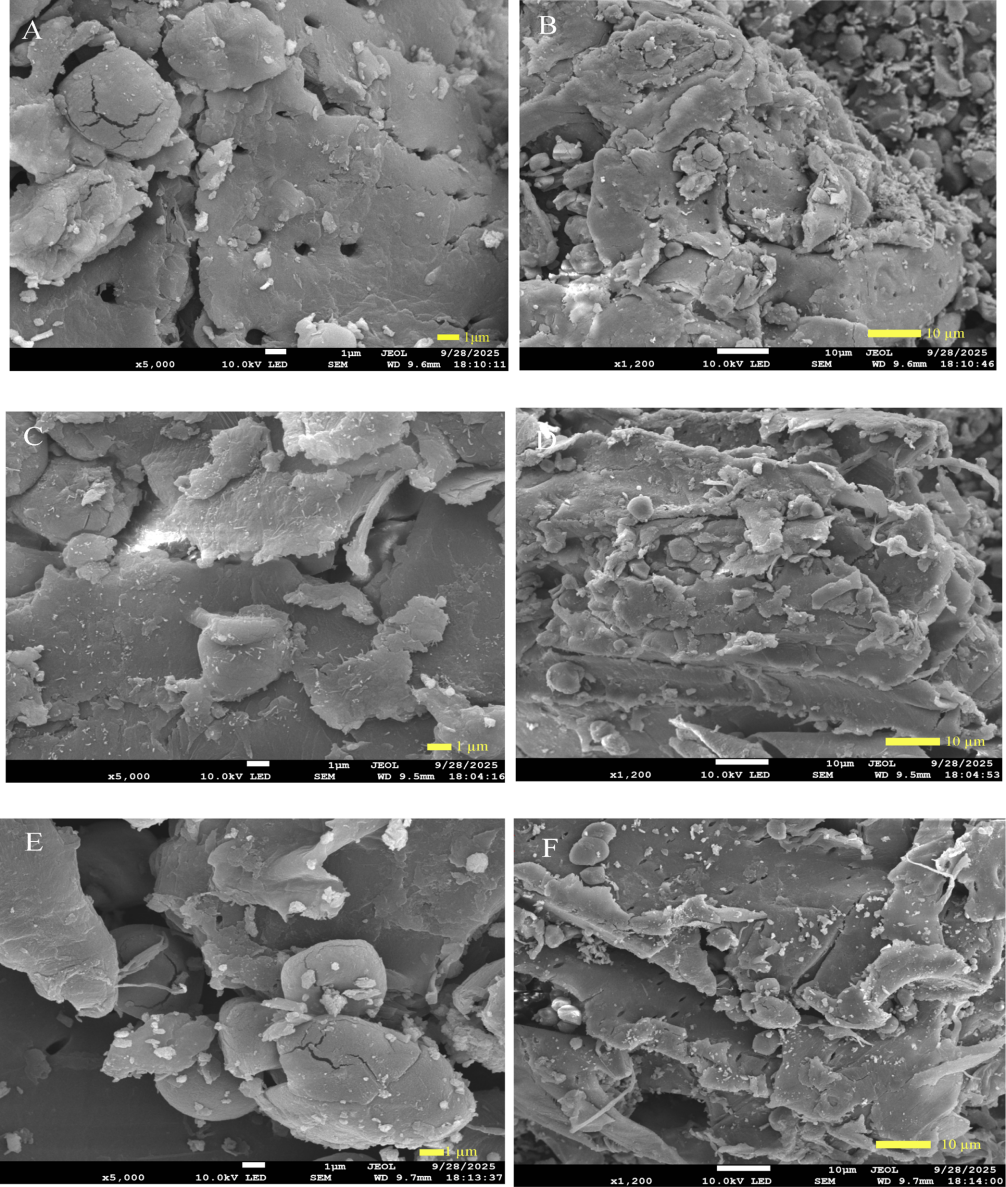
Scanning electron microscopy analysis of *P. cuspidatum*. **A** (×5000), **B** (×1200) The original powder, **C** (×5000), **D** (×1200) extraction with DES9, and **E** (×5000), **F** (×1200) ultrasound-assisted extraction with DES9.

#### 3.5.2 Fourier Transform Infrared Spectroscopy Analysis

As a sophisticated analytical technique, FT-IR utilized characteristic infrared absorption wavelengths and intensities to identify the functional groups of substances. It is thus extensively applied to confirm molecular interactions between different molecules.^32^ As described in Figure 5A, the stretching vibrations of the −C–O and −OH groups of Gly were observed at the characteristic absorption peaks of 1028.8 and 3287.6 cm^–1^, respectively. For Bet, the absorption peaks at 1620.3 and 1384.1 cm^–1^ corresponded to the antisymmetric stretching vibration of −C=O and the stretching vibrations of −CH_3_ groups, respectively. Compared with those of pure Bet and Gly, the significant blue shift of the Gly absorption peak in DES9 indicated the formation of intermolecular H-bonds within DES9. For instance, the −OH stretching vibration peak of Gly showed a notable downfield shift (3287.6 to 3302.9 cm^–1^, Δδ = 15.3 cm^–1^). Consistent with prior reports,^35^ these FT-IR results confirmed the successful preparation of DES9. After the addition of PLD, the broad 3290.9 cm^–1^ absorption peak in the DES9 extract spectrum was notably narrowed, directly indicating that PLD altered the H-bonding network of DES9 during the extraction process. These findings further verify that the DES extraction process was a physical process, which conferred significant advantages for the subsequent recovery and reuse of DESs.

**Figure 5:**
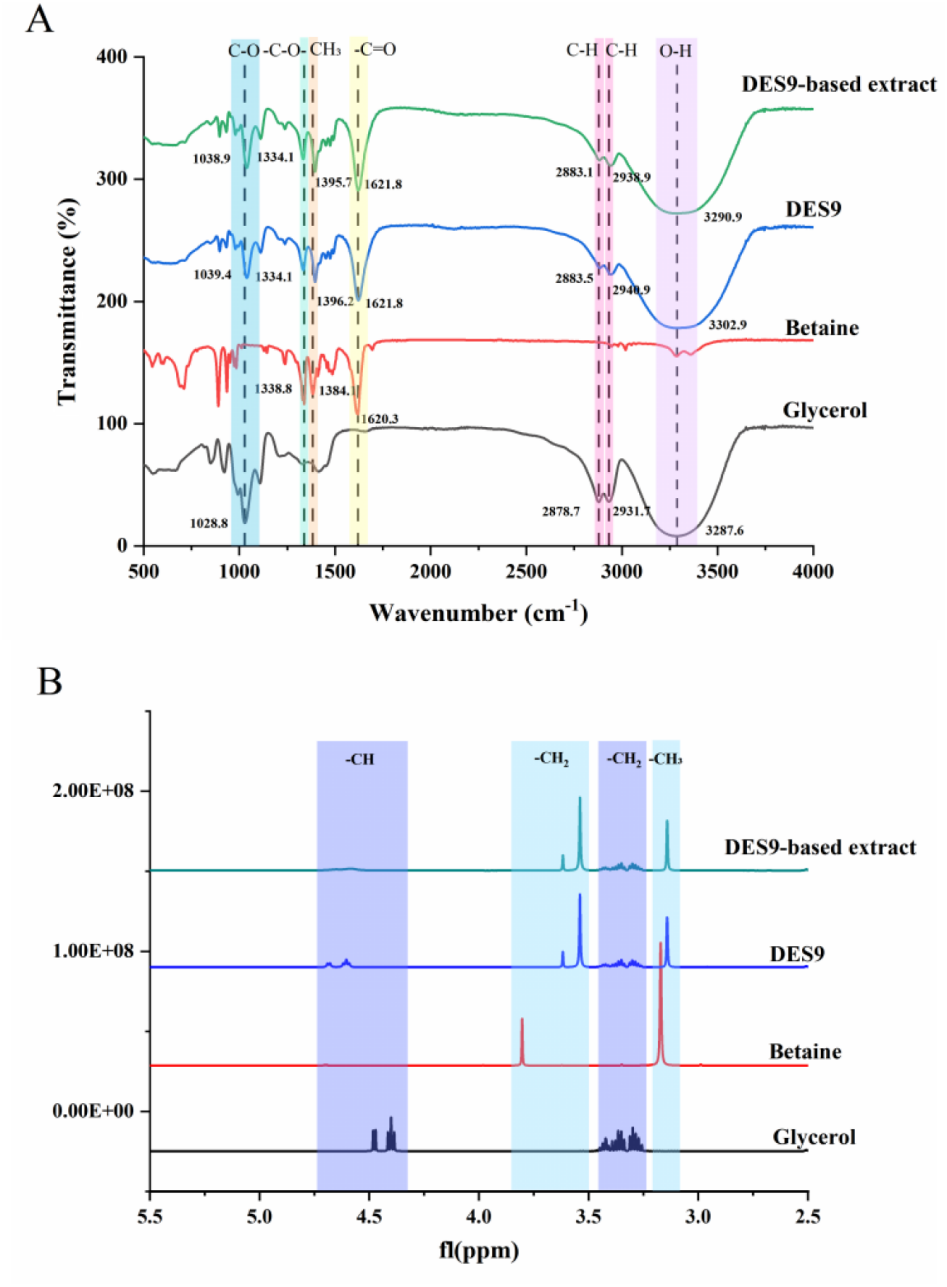
Characterization of the DES9-based extract. **A** FT-IR spectrogram and **B** ^1^H NMR spectrogram.

#### 3.5.3 Nuclear Magnetic Resonance Hydrogen Spectrum Analysis

The chemical shift variations of hydrogen protons can directly reflect the microenvironmental changes of molecules during the DES extraction process.^36^ As illustrated in Figure 5B, the distinctive peaks at 3.8 and 3.17 ppm belonged to the −CH_2_ and −CH_3_ protons of Bet, respectively, while the characteristic peak at 4.43 ppm was assigned to the −CH proton of Gly. In DES9, the −CH proton of Gly showed a notable downfield shift (4.43 to 4.64 ppm, Δδ = 0.21 ppm), whereas the −CH_2_ protons of Bet displayed an upfield shift (3.8 to 3.54 ppm, Δδ = 0.34 ppm). Hence, with the formation of H-bonds between Bet and Gly in DES9, the shielding effect exerted by the −COO^–^ groups of Bet on the adjacent H-bond protons of −CH_2_ was attenuated. Notably, the ^1^H NMR spectrum of the DES9 extract showed no discernible changes following the addition of PLD. The proton peaks corresponding to Bet and Gly remained clearly distinguishable in the DES9 extract, indicating that the molecular functional groups within the DES9 extract maintained structural stability. These findings further verify that the PLD extraction process using DES9 was a physical process, which conferred significant advantages for the subsequent recovery and reuse of DESs.

#### 3.5.4 Molecular Dynamics Analysis

Molecular dynamics was a computational simulation approach based on the principles of Newtonian mechanics. It provided profound insights into the behavior of complex extraction processes, such as binding energy and H-bonding between molecules.^34^ The amorphous cell of the DES9 extract was exhibited in Figure 6A. It can be observed that the majority of PLD resided at the periphery of the amorphous cell, while a small number of PLD were embedded within the DES9 domains in the interior. This dissolution pattern further highlighted the hydrophobic nature of the PLD.

**Figure 6:**
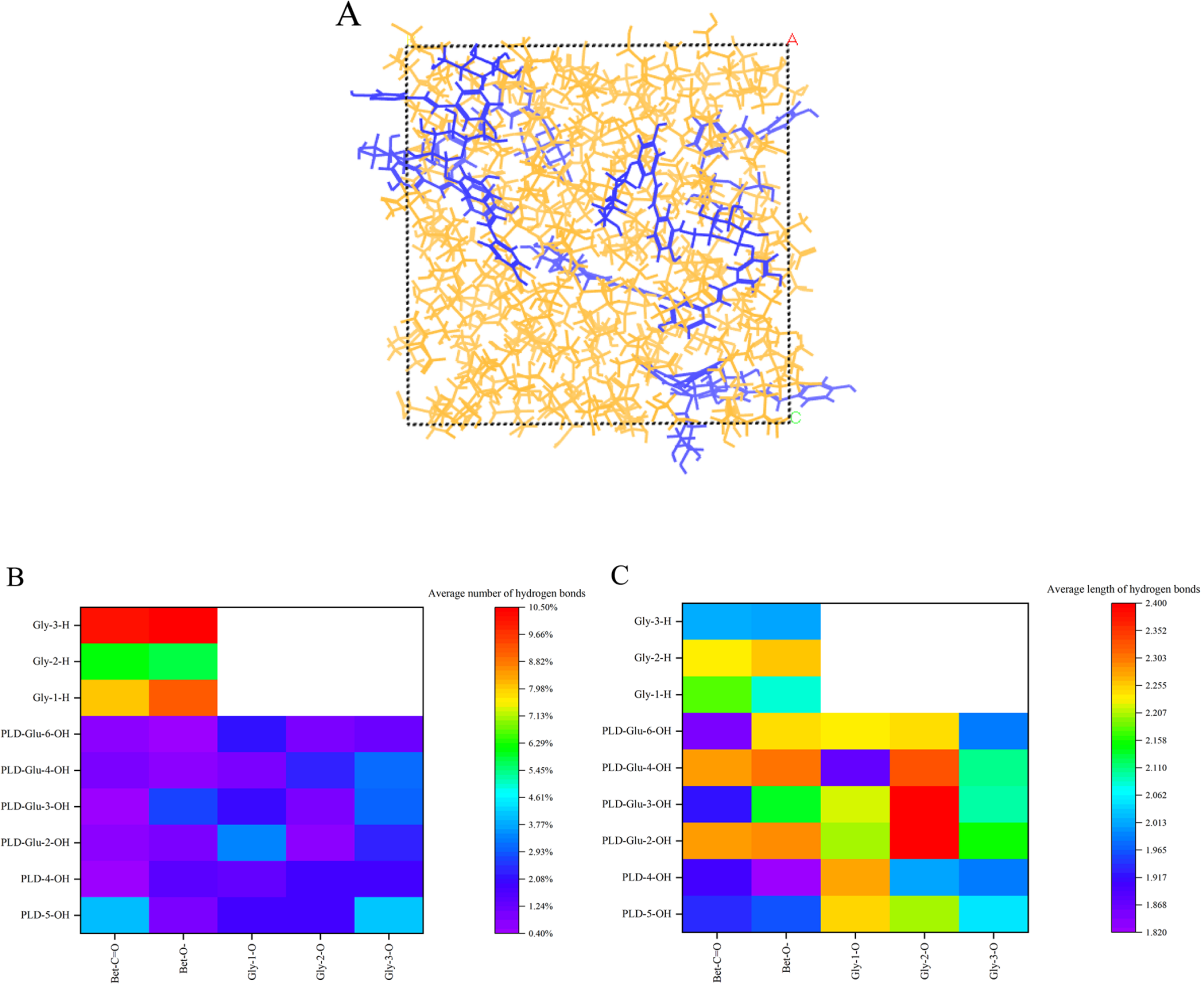
Results of the molecular dynamics of the DES9 extract. **A** The amorphous cell construction of the DES9 extract, **B** the average number of hydrogen bonds, and **C** the average length of hydrogen bonds.

The binding energy, as a thermodynamic descriptor of structural stability, was more negative when the target compound bound to the extractant with a stronger affinity. And the smaller the negative binding energy, the greater the extraction efficiency and enrichment.^34^ The binding energy of PLD among the selected DESs is exhibited in Table 3. Compared with the Bet–Gly binding energy in DES9, it increased to −994.72 kcal/mol after PLD extraction. The results implied that the incorporation of PLD disrupted the native binding architecture between Gly and Bet, diminishing their mutual interaction energy. The binding energy between PLD and DES9 was lower than that of other DES extracts, with a value of −994.72 kcal/mol. Hence, DES9 possessed superior extraction capabilities for the PLD.

**Table 3:** Interaction Energy within DES9, and between PLD and 5 Candidates DESs

DESs	Eint (kcal/mol)	Elec in Eint (kcal/mol)	vdW contribution (kcal/mol)
DES9	–1240.73	–789.38	–451.34
DES9-PLD	–994.72	–647.30	–347.42
DES6-PLD	–909.11	–543.49	–365.62
DES4-PLD	–983.83	–678.31	–305.52
DES1-PLD	–932.92	–585.50	–347.43
DES7-PLD	–993.68	–594.96	–398.71

Electrostatic attraction was the primary driving force for hydrogen bond formation, which constituted a pivotal mechanism governing the functional properties of DESs. Through the rational screening of HBDs and HBAs with substantial electrostatic potential disparities, the H-bond density and physicochemical characteristics of DESs can be systematically regulated, ultimately enhancing their extraction efficiency.^20^ In the extraction process, electrostatic interactions of the DES9 extract were the predominant force, characterized by the lowest interaction energy value of −647.30 kcal/mol. The heatmap of the average number of H-bonds was depicted in Figure 6B. There were several H-bonding interactions between Bet and Gly, especially Bet-O^–^···3-OH-Gly, with the highest proportion of 10.47%. Notably, the interaction between PLD and DES9 involved numerous H-bonds. In contrast to the interaction between PLD and Bet, the number of H-bonds formed between PLD and Gly was greater with a proportion of 36.05%. The results were in agreement with the prior theoretical analysis of COSMO-RS. The HBD role of PLD contributed to the enhancement of the binding affinity. For example, PLD-5-OH···OH-Gly had a proportion of 7.87%, PLD-4-OH···OH-Gly had a proportion of 5.08%, and PLD-Glu-OH···OH-Gly had a proportion of 23.10%. Therefore, Gly had dual identities, acting as both the HBA and HBD. PLD, acting as an HBD, was partially solubilized by competitively displacing Gly from its native HBD role within DES. This competitive substitution not only disrupted the original H-bond network in DESs but also established new intermolecular interactions between PLD and DES components.

The average length of H-bonds between PLD and DES9 in the range of 1.8–2.5 nm is also calculated and is shown in Figure 6C. Compared to Gly, the H-bonds between PLD and Bet were more stable, as evidenced by their shorter average length. For example, PLD-4-OH···C=O-Bet with an average length of 1.90 nm, PLD-Glu-3-OH···C=O-Bet with an average length of 1.92 nm, and PLD-5-OH···O-Bet with an average length of 1.96 nm. The outcomes further confirmed the role of PLD as an HBD and provided robust mechanistic support for the competitive binding behavior of PLD and Gly toward HBA in the complex extraction process. Additionally, the distinct interaction tendency of PLD with Gly exhibited strong consistency with the thermodynamic characteristics of HBDs elucidated by COSMO-RS. Hence, an excessively high molar ratio was deemed unfavorable, given the relatively weak nature of the interaction between PLD and Gly.

### 3.6 The Stability of PLD in Deep Eutectic Solvents

As an inherently unstable molecule, PLD is susceptible to degradation upon exposure to light, heat, or moisture, necessitating strict low-temperature and light-shielded storage conditions throughout all experimental procedures.^3^ DESs functioned as dual-purpose agents, serving both as cosolvents and stabilizers, thus effectively mitigating the degradation of labile bioactive molecules.^13^ The stability of PLD in DES9 is also pivotal for practical application prospects. As illustrated in Figure S4-A, the total degradation rate of PLD in DES9 was determined to be 10.07%, with the most rapid degradation occurring within the initial 20 days, accounting for 10.04% of the total loss. Thereafter, the PLD concentration in DES9 exhibited negligible fluctuations from day 20 to day 60, maintaining a relatively steady level. In stark contrast, PLD degraded much more rapidly in an ethanol solution, with its degradation degree reaching 71.34% after 60 days of storage. As illustrated in Figure S4-B, the degradation rate of PLD in DES9 stored at 80 °C was minimal at 4.23%, while the degradation rate of PLD stored at 100 and 120 °C was increased. These results suggested that temperature plays an important role in maintaining the stability of PLD. Similarly, PLD exhibited the most significant degradation in ethanol solution, with its degradation degree reaching 11.12%, 30.56%, and 41.67% at 80 °C, 100 °C, and 120 °C, respectively. This marked discrepancy in stability may be ascribed to the hydrogen-bonding interactions formed between the PLD and DES9 components. Collectively, DES9 acted as both an efficient extractant and a stabilizer, which not only facilitated the extraction of PLD but also endowed the resultant DES extract with favorable stability, thereby supporting its potential development as an active additive.

### 3.7 The Antioxidant Activity

DES-based extracts were a valuable and biologically compatible medium for the production of effective films. The antioxidant activity of the DES-based extract was highly dependent on the plant extract. DPPH was commonly used to investigate the antioxidant activity of natural products.^37^ As exhibited in Figure S5-A, the DES9-based extract exhibited a concentration-dependent DPPH radical scavenging effect with an IC_50_ of 246.38 μg/mL, which corresponds to the PLD concentrations of 5.08 μg/mL. Hydroxyl radicals, a well-recognized inducer of biological macromolecule damage, are closely correlated with the pathophysiological processes of oxidative stress.^37^ As exhibited in Figure S5-B, the DES9-based extract exhibited a concentration-dependent hydroxyl radical scavenging effect with an IC_50_ of 2.19 mg/mL, which corresponds to the PLD concentrations of 45.21 μg/mL. Compared with the ethanol extract, the DES9-based extract also exhibited superior antioxidant efficacy. This enhancement was likely attributable to the high abundance of PLD. Notably, DESs formulated with Bet and Gly as components have been widely employed in gelatin-based active packaging, flue gas desulfurization gel, and a biopolymeric sensor for monitoring meat spoilage.^38^ Therefore, the DES9-based extract was a promising candidate for use as an antioxidant supplement for functional materials.

### 3.8 The Cytotoxicity Assay

PLD, as the main active ingredient of *P. cuspidatum*, possessed a diverse array of biological activities, including antioxidant, neuroprotective, antitumor, cardioprotective, and metabolic regulatory properties.^1–3^ DESs have additionally received significant attention for versatile applications, as their customizable properties render them highly promising for utilization.^39^ However, the cytotoxicity of the DES-based extract may differ from its individual components. This discrepancy could be ascribed to the synergistic interactions among the constituent components. Hence, the potential toxicity of the DES-based extract should not be overlooked. As illustrated in Figure S6, HaCaT cell viability was enhanced by both DES9 and the DES9-based extract. A distinct dose-dependent effect was observed across the concentration range of 10 to 40 mg/mL. In comparison with DES9, the DES9-based extract exhibited a superior viability increase. The most significant improvement in cell viability was elicited by the DES9-based extract at 40 mg/mL, where the viability value approached nearly 151.6%. Hence, there was a synergistic effect between DES9 and PLD in promoting cell proliferation. These results are consistent with relevant literature reports demonstrating that PLD and DES9 can promote the proliferation of skin fibroblasts.^1–3^ At concentrations ranging from 80 to 320 mg/mL, despite a reduction in cellular viability, the cells remained highly viable. This finding strongly confirms that both DES9 and DES9-based extract formulations are noncytotoxic, which provides robust support for their potential as safe candidates for diverse biomedical and transdermal applications.

## 4 Conclusion

In the present study, an efficient UA-DES extraction method was developed and validated for the targeted extraction of PLD from *P. cuspidatum*. Guided by COSMO-RS, Bet and Gly were selected as the optimal HBA and HBD, respectively. The experimental results for PLD extraction were in good agreement with the predicted solubility trends of PLD in various DESs. Accordingly, the optimized DES formulated with Bet and Gly at a molar ratio of 2:1 and 40 wt % water was selected for subsequent extraction experiments. And the optimized extraction parameters were determined as 28:1 mL/g of liquid–material ratio, 54 min of extraction duration, and 246 W of ultrasonic power, under which the PLD yield reached 20.34 ± 0.55 mg/g. Notably, the superior extraction efficiency of UA-DES9 was tightly correlated to its intrinsic capacity to disrupt the structural integrity of *P. cuspidatum* cell walls. Furthermore, the extraction mechanism was explored by FT-IR and ^1^H NMR, and molecular dynamics simulations. These results demonstrated that electrostatic interactions within DES9 constitute the primary driving force for the efficient extraction of PLD, with a minimum interaction energy of −994.72 kJ/mol. Additionally, H-bonding interactions between PLD and DES9 were observed. PLD functioned as an HBD, engaging in competitive interactions with the HBA sites of Bet. And PLD formed H-bonds with Gly, which displayed the dual traits of high density and intrinsically weak binding force. These findings have substantially deepened the fundamental understanding of the extraction mechanism inherent to UA-DES, offering strong cross-validation for the thermodynamic property analysis of PLD and DESs derived from COSMO-RS. Moreover, the DES9 extract exhibited nontoxicity, excellent antioxidant activity, and favorable stability, making it a promising candidate as an antioxidant supplement for functional materials. This study not only provided a robust theoretical foundation for the rational design and sustainable application of DESs in plant extraction but also furnished novel insights into advancing green, efficient technologies for plant resource utilization and ecological sustainability.

## Supplementary Material



## Data Availability

Data will be made available on request.

## Notes

Experimental setup for the measurement of density, the stability of PLD in DESs; the antioxidant activity, the cytotoxicity assay; (Table S1) The structure of polydatin, hydrogen bond acceptors, and hydrogen bond donors; (Table S2) The categories of deep eutectic solvents; (Table S3) The design of experimental levels for the molar ratio, water content, and single-factor experiments; (Table S4) The design of experimental levels for Box–Behnken design; (Table S5) Parameter settings for the simulation system model; (Table S6) The σ-profile location of hydrogen bond donors; (Table S7) The σ-profile location of deep eutectic solvents; (Table S8) Experimental and MS predictive densities at 50 °C for DESs; (Figure S1) The σ-profile and σ-potential diagrams of deep eutectic solvents; (Figure S2) The results of single-factor experiment: (A) extraction time, (B) liquid–material ratio, (C) extraction time, and (D) ultrasonic power; (Figure S3) Predicted vs actual plots; (Figure S4) The stability of PLD in deep eutectic solvents and in ethanol; (Figure S5) Results of antioxidant experiments: (A) the DPPH free radical scavenging ratio and (B) the hydroxyl radical scavenging ratio; (Figure S6) Cell viability of DES9-based extract and DES9 for HaCaT cells; (Figure S7) The HPLC chromatogram of polydatin standard solution, DES9, ethanol, and water extract; (Figure S8–S12) Molecular dynamics simulation of DES-PLD (PDF)
