# Supplementary material for: Molecular Design and Experimental Study of Deep Eutectic Solvents Extraction of Polydatin from Polygonum cuspidatum
Source: ACS Omega. 2026 Apr 13;11(16):24665–78. doi: 10.1021/acsomega.6c01051 (PMC13129837; doi:10.1021/acsomega.6c01051)
Supplement: Supplementary file 1 [file ao6c01051_si_001.pdf]

Electronic Supplementary Material

Molecular design and experimental study of deep eutectic solvents extraction of polydatin from *Polygonum cuspidatum*

Xu Cai[1,2#], Yuanguo Xiong [1#], Zhouwei Hong[3], Tong Xia[3], Dan Li[1*], Liu Tang[1*]

[1]Department of Pharmacy, Renmin Hospital of Wuhan University, Wuhan Hubei 430000, P.R.China.

[2]Department of Engineering Physics, Tsinghua University, Beijing 100084, P.R.China

[3]College of Pharmacy, Hubei University of Chinese Medicine, Wuhan Hubei 430000, P.R.China.

[#]Co-first author.

*Corresponding author**:** Dan Li, Department of Pharmacy, Renmin Hospital of Wuhan University, Wuhan Hubei 430000, P.R.China.

Email: 15926329308@163.com

Additional corresponding author

Liu Tang

Email: 616112135@qq.com

1.1 Measurement of the density

The relative density of DESs was determined on the basis of the pycnometer method. Initially, the pycnometer was successively cleaned with acetone, anhydrous ethanol, and ultrapure water using ultrasonication, and then dried at 105 ℃. After that, the weighed of empty pycnometer was recorded as W_0. Subsequently, the pycnometer was filled with distilled water and DESs solution, respectively. Then it was placed in 25 ℃ water for 15 minutes to equilibrate and eliminate air bubbles. After wiping the outer surface dry, the pycnometer was weighed to obtain the masses of the pycnometer and water (W_1), the pycnometer and DES (W_2). The relative density of DESs was calculated as Formula (3):

$$\rho = \frac{W_1 - W_0}{W_2 - W_0} \tag{3}$$

where ρ represents the relative density of DES.

1.2 The stability of PLD in DESs

The impact of storage duration on PLD in DES9 was investigated. DES9-based extracts were transferred into brown glass bottles and stored at 25℃ for 0, 20, 40, and 60 days, respectively. In addition, DES9-based extracts were incubated at 80, 100, and 120℃ for 8.0 h. The degradation rate was calculated as $(C_0\text{-}C)/C_0 \times 100\%$, where C_0 was the initial PLD concentration, and C was the PLD concentration after storage.

1.3 The antioxidant activity

1.3.1 DPPH free radicals scavenging experiment

Ethanol solution of DES9 extract (32.4, 16.2, 8.1, 4.05, 2.03, 1.01, 0.51, and 0.25 μg/mL) was reacted with 0.1 mmol/L DPPH solution in equal volumes. The reaction solution was

carried out at 25°C for 30 min, after which the absorbance was measured by Synergy H1 microplate reader (BioTek Instruments, Inc., Vermont, USA) at 517 nm. The scavenging ratio of DPPH free radicals (Y_3, %) was calculated as Formula (4):

$$Y_3 = \frac{A_1 - (A_2 - A_3)}{A_1} \times 100\% \tag{4}$$

Where A_1: the absorbance of the blank solution, A_1: the absorbance of test solution, A_2: the absorbance of sample solution.

1.3.2. Hydroxyl radicals scavenging experiment

Ethanol solution of DES9 extract (402, 161.4, 80.7, 40.35, 20.18, 10.09, 5.04, and 2.52 μg/mL) was mixed with 1.8 mmol/L ferrous sulphate solution and salicylic acid-ethanol solution. Then, 0.03 wt% the hydrogen peroxide was added. The reaction solution was carried out at 25°C for 30 min, after which the absorbance was measured by Synergy H1 microplate reader (BioTek Instruments, Inc., Vermont, USA) at 510 nm. The scavenging ratio of hydroxyl radical (Y_4, %) was calculated as Formula (5):

$$Y_4 = \frac{A_1 - A_0}{A_2 - A_0} \times 100\% \tag{5}$$

Where A_0: the absorbance of the negative solution; A_1: the absorbance of sample solution, A_2: the absorbance of blank solution.

1.4 The cytotoxicity assay

The cytotoxicity of DES9 extract toward human keratinocyte (HaCaT) cells was assessed by the MTT assay. HaCaT cells were seeded into 96-well plates and incubated at 37°C with 5% CO_2 until fully adherent was then added. And the plates were incubated for an additional 24 h before viability measurement. Subsequently, the supernatant was gently removed from each well. After that, 90 μL DMEM medium and 10 μL MTT solution were added, respectively. After 4 hours of incubation, the culture was terminated. The supernatant was carefully removed, and the cells were gently washed with PBS. To dissolve the formazan

crystals, added 100 μL of DMSO to each well. Finally, the absorbance value of each well was measured by Synergy H1 microplate reader (BioTek Instruments, Inc., Vermont, USA) at 570 nm. The cell viability (Y_5, %) was calculated as Formula (6):

$$Y_5 = \frac{A_1 - A_0}{A_2 - A_0} \times 100\% \quad (6)$$

Where A_0: the absorbance of DMSO, A_1: the absorbance of samples, A_2: the absorbance of control.

2. Results and Discussion

Before the solubility screening, the density (ρ) of DESs at 50℃ was predicted in MS, and the results are presented in Table S8. The goal was to compare the results with already published values to verify if the approach chosen to represent the DESs was accurate. The small deviations were observed. As MS is a fully predictive model that only considers the molecular structure of each compound, an error of less than 5% was considered acceptable for the approach chosen. Hence, the MS model is an accurate tools in selecting an appropriate extraction solvent for PLD.

Table of Contents

CONTENTS	page
**Table S1.** The structure of polydatin, hydrogen bond acceptors and hydrogen bond donors	6
**Table S2.** The categories of deep eutectic solvents	7
**Table S3.** The design of experimental levels for the molar ratio, water content and single-factor experiments	7
**Table S4.** The design of experimental levels for Box-Behnken design	8
**Table S5.** Parameter settings for the simulation system model	8
**Table S6.** The σ-profile location of hydrogen bond donors	8
**Table S7.** The σ-profile location of deep eutectic solvents	9
**Table S8**. Experimental and MS predictive densities at 50℃ for DESs	9
**Figure S1.** The σ-profile and σ-potential diagrams of deep eutectic solvents	10
**Figure S2.** The results of single factor experiment. (A): Extraction time; (B): Liquid-material ratio; (C): Extraction time; (D): Ultrasonic power.	11
**Figure S3**. Predicted vs. actual plots	11
**Figure S4.** The stability of PLD in deep eutectic solvents and in ethanol.	12
**Figure S5.** Results of antioxidant experiments. (A): The DPPH free radical scavenging ratio; (B) The hydroxyl radical scavenging ratio.	12
**Figure S6.** Cell viability of DES9-based extract and DES9 for HaCaT cells	12
**Figure S7**. The HPLC chromatogram of polydatin standard solution, DES9, ethanol, and water extract	13
**Figure S8** Molecular dynamics simulation of BET-Gly-PLD	14
**Figure S9** Molecular dynamics simulation of BET-LA-PLD	15
**Figure S10** Molecular dynamics simulation of BET-MA-PLD	16
**Figure S11** Molecular dynamics simulation of ChCl-LA-PLD	17
**Figure S12** Molecular dynamics simulation of ChCl-Gly-PLD	18

Table S1 The structure of polydatin, hydrogen bond acceptors and hydrogen bond donors.

Type	Name		Structural formula
**Target compound**	Polydatin	PD	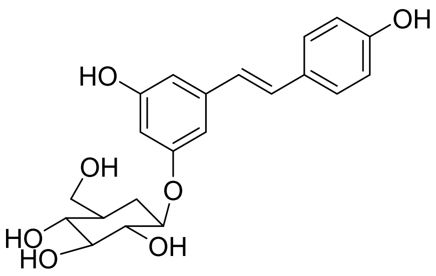
**HBA**	Choline chloride	ChCl	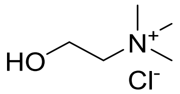
	Betaine	BET	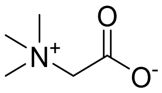
**HBD**	Lactic acid	LA	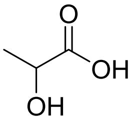
	Malic acid	MA	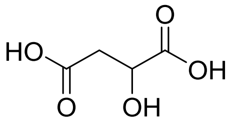
	Citric acid	CA	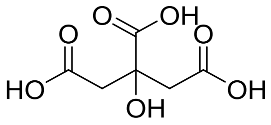
	Glycerol	Gly	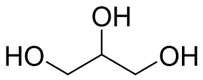
	Glucose	Glu	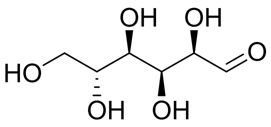
	Ascorbic acid	AA	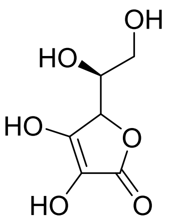

Fructose	Fru	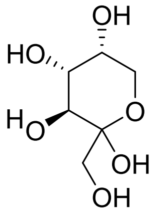
Sucrose	Suc	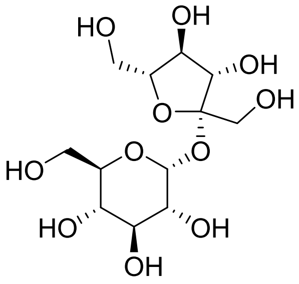
Tartaric Acid	TA	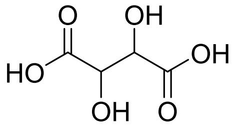
Xylitol	Xyl	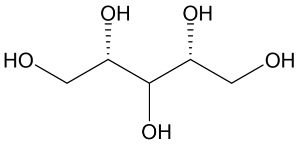

Table S2 The categories of deep eutectic solvents

Group	HBD	HBA	Molar ratio	Group	HBD	HBA	Molar ratio
DES1	LA			DES11	AA		
DES2	MA			DES12	Fru		
DES3	CA	ChCl		DES13	Suc	ChCl	
DES4	Gly			DES14	TA		
DES5	Glu			DES15	Xyl		
DES6	LA		2:1	DES16	AA		2:1
DES7	MA			DES17	Fru		
DES8	CA	Bet		DES18	Suc	Bet	
DES9	Gly			DES19	TA		
DES10	Glu			DES20	Xyl		

Table S3 The design of experimental levels for the molar ratio and water content

Factors	Unit	Levels				
The molar ratio		1:2	1:1	2:1	3:1	4:1
Water content	wt%	30	40	50	60	70
Extraction time	min	10	20	40	60	80

Liquid-material ratio	mL/g	10:1	20:1	30:1	40:1	50:1
Ultrasonic power	W	100	150	200	250	300
Extraction temperature	℃	30	40	50	60	70

Table S4 The design of experimental levels for Box-Behnken design

Factors	Unit	Code	Levels		
			-1	0	1
Extraction time	min	A	40	60	80
Liquid-material ratio	mL/g	B	20:1	30:1	40:1
Ultrasonic power	W	C	200	250	300

Table S5 Parameter settings for the simulation system model

The Amorphous Cell	Number of molecules					AC dimensions
	HBA	HBD	Water	PLD	Ethanol	
Bet-Gly-PLD	50	100	-	10	-	30.1 Å× 30.1 Å× 30.1 Å
Bet-LA-PLD	50	100	-	10	-	30.1 Å× 30.1 Å× 30.1 Å
ChCl-Gly-PLD	50	100	-	10	-	30.9 Å× 30.9 Å× 30.9 Å
ChCl-LA-PLD	50	100	-	10	-	30.9 Å× 30.9 Å× 30.9 Å
Bet-MA-PLD	50	100	-	10	-	31.3 Å× 31.3 Å× 31.3 Å
H_2O-PLD	-	-	100	10	-	21.2 Å× 21.2 Å× 21.2 Å
ET-PLD	-	-	30	10	70	25.2 Å×25.2 Å× 25.2 Å

Table S6 The σ-profile location of hydrogen bond donors

Hydrogen bond donors	HBD	Non-polar	HBA
LA	-0.023 e/Å^2 <σ< -0.0084 e/Å^2		0.0084 e/Å^2 < σ< 0.019 e/Å^2
MA	-0.023 e/Å^2 <σ< -0.0084 e/Å^2		0.0084 e/Å^2 < σ< 0.016 e/Å^2
CA	-0.025 e/Å^2 <σ< -0.0084 e/Å^2		0.0084 e/Å^2 < σ< 0.016e/Å^2
Gly	-0.020 e/Å^2 <σ<-0.0084 e/Å^2		0.0084 e/Å^2 < σ< 0.021 e/Å^2
Glu	-0.021 e/Å^2 <σ< -0.0084 e/Å^2	-0.0084 e/Å^2 < σ<	0.0084 e/Å^2 < σ< 0.020 e/Å^2
AA	-0.021 e/Å^2 <σ<-0.0084 e/Å^2	0.0084 e/Å^2	0.0084 e/Å^2 < σ < 0.017e/Å^2
Fru	-0.021e/Å^2 <σ<-0.0084 e/Å^2		0.0084 e/Å^2 < σ < 0.020e/Å^2
Suc	-0.020e/Å^2 <σ<-0.0084 e/Å^2		0.0084 e/Å^2 < σ < 0.026e/Å^2
TA	0.023e/Å^2 <σ<-0.0084 e/Å^2		0.0084 e/Å^2 < σ < 0.017e/Å^2
Xyl	-0.020e/Å^2 <σ<-0.0084 e/Å^2		0.0084 e/Å^2 < σ < 0.021e/Å^2

Table S7 The σ-profile location of deep eutectic solvents

Deep eutectic solvents	HBD	Non-polar	HBA
DES1	-0.023 e/Å^2 <σ< -0.0084 e/Å^2		0.0084 e/Å^2 < σ< 0.020 e/Å^2
DES2	-0.023 e/Å^2 <σ<-0.0084 e/Å^2		0.0084 e/Å^2 < σ< 0.020 e/Å^2
DES3	-0.021 e/Å^2 <σ<-0.0084 e/Å^2		0.0084 e/Å^2 < σ< 0.019 e/Å^2
DES4	-0.020 e/Å^2 <σ<-0.0084 e/Å^2		0.0084 e/Å^2 < σ<0.021 e/Å^2
DES5	-0.022 e/Å^2 <σ<-0.0084 e/Å^2		0.0084 e/Å^2 < σ<0.019 e/Å^2
DES6	-0.023 e/Å^2 <σ< -0.0084 e/Å^2		0.0084 e/Å^2 < σ< 0.025 e/Å^2
DES7	-0.022 e/Å^2 <σ<-0.0084 e/Å^2		0.0084 e/Å^2 < σ< 0.018 e/Å^2
DES8	-0.023 e/Å^2 <σ< -0.0084 e/Å^2		0.0084 e/Å^2 < σ< 0.021e/Å^2
DES9	-0.023 e/Å^2 <σ< -0.0084 e/Å^2	-0.0084 e/Å^2 < σ<	0.0084 e/Å^2 < σ< 0.025 e/Å^2
DES10	-0.021 e/Å^2 <σ< -0.0084 e/Å^2	0.0084 e/Å^2	0.0084 e/Å^2 < σ<0.025 e/Å^2
DES11	-0.023e/Å^2 <σ<-0.0084 e/Å^2		0.0084 e/Å^2<σ< 0.020e/Å^2
DES12	-0.024e/Å^2 <σ<-0.0084 e/Å^2		0.0084 e/Å^2<σ< 0.020e/Å^2
DES13	-0.025e/Å^2 <σ<-0.0084 e/Å^2		0.0084 e/Å^2<σ< 0.024e/Å^2
DES14	-0.020e/Å^2 <σ<-0.0084 e/Å^2		0.0084 e/Å^2<σ< 0.020e/Å^2
DES15	-0.019e/Å^2 <σ<-0.0084 e/Å^2		0.0084 e/Å^2<σ< 0.021e/Å^2
DES16	-0.023e/Å^2 <σ<-0.0084 e/Å^2		0.0084 e/Å^2<σ< 0.025e/Å^2
DES17	-0.024e/Å^2 <σ<-0.0084 e/Å^2		0.0084 e/Å^2<σ< 0.025e/Å^2
DES18	-0.025e/Å^2 <σ<-0.0084 e/Å^2		0.0084 e/Å^2<σ< 0.026e/Å^2
DES19	-0.023e/Å^2 <σ<-0.0084 e/Å^2		0.0084 e/Å^2<σ< 0.025e/Å^2
DES20	-0.021e/Å^2 <σ<-0.0084 e/Å^2		0.0084 e/Å^2<σ< 0.025e/Å^2

Table S8. Experimental and MS predictive densities at 50℃ for DESs

DES	Density (g/mL)			
	Average	Std. Dev.	Experimental	Error (%)
BET-Gly-PLD	1.181	0.027	1.165	-1.35
BET-LA-PLD	1.196	0.035	1.189	-0.58
BET-MA-PLD	1.303	0.034	1.312	0.69
ChCl-LA-PLD	1.045	0.030	1.028	-1.62
ChCl-Gly-PLD	1.043	0.027	1.036	-0.67

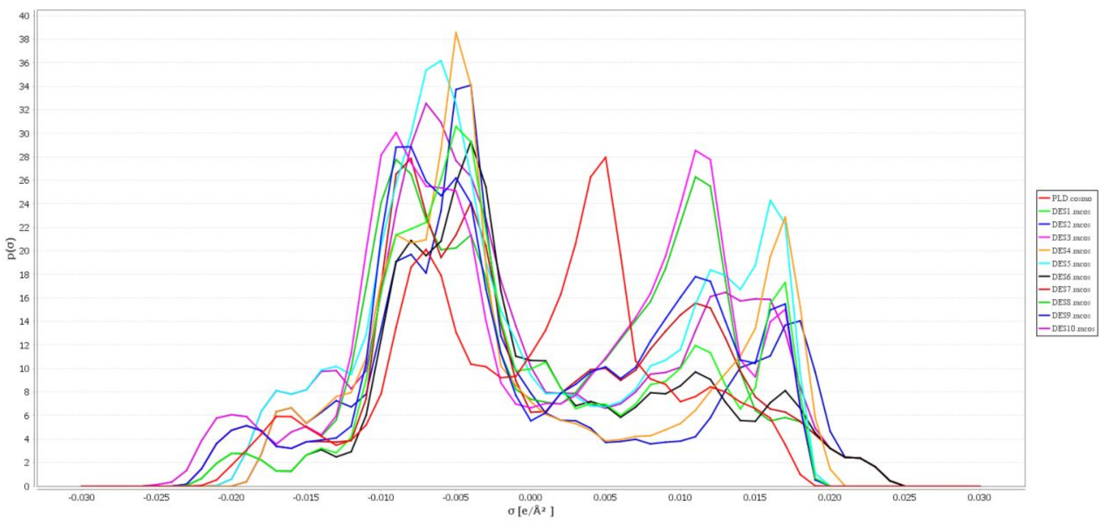
p(σ)
σ [e/Å²]
PLD.cosmo
DES1.mcos
DES2.mcos
DES3.mcos
DES4.mcos
DES5.mcos
DES6.mcos
DES7.mcos
DES8.mcos
DES9.mcos
DES10.mcos

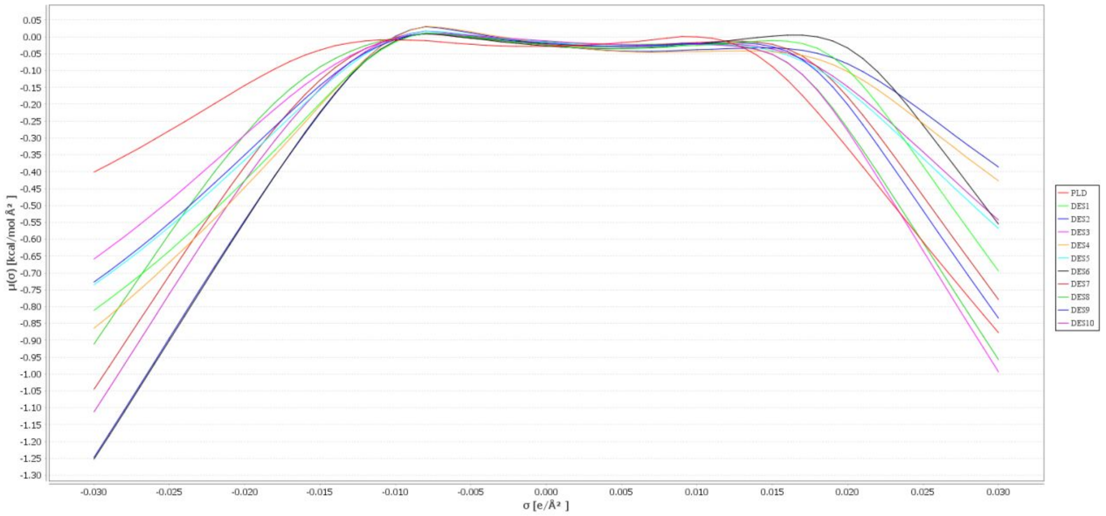
μ(σ) [kcal/mol Å²]
σ [e/Å²]
PLD
DES1
DES2
DES3
DES4
DES5
DES6
DES7
DES8
DES9
DES10

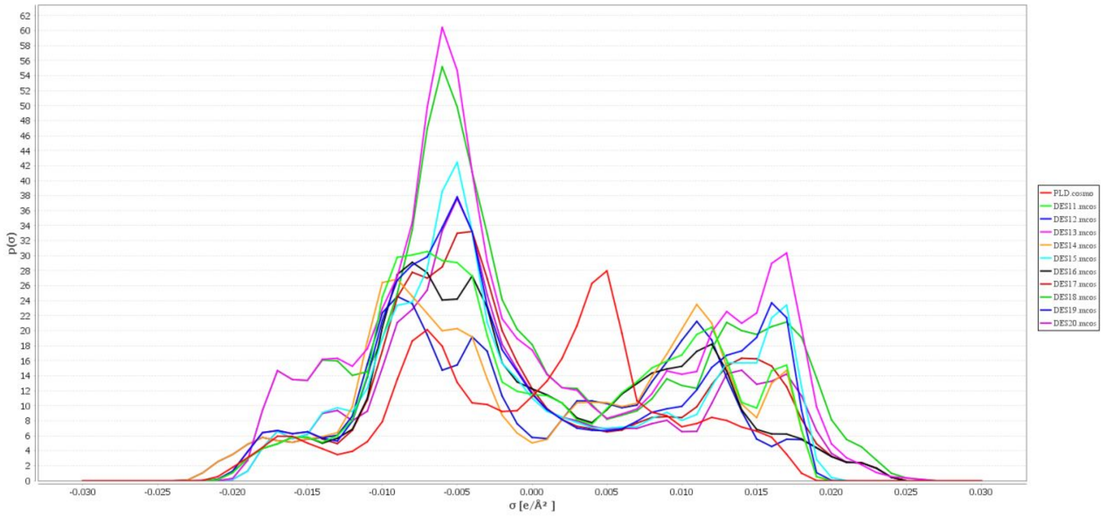
p(σ)
σ [e/Å²]
PLD.cosmo
DES11.mcos
DES12.mcos
DES13.mcos
DES14.mcos
DES15.mcos
DES16.mcos
DES17.mcos
DES18.mcos
DES19.mcos
DES20.mcos

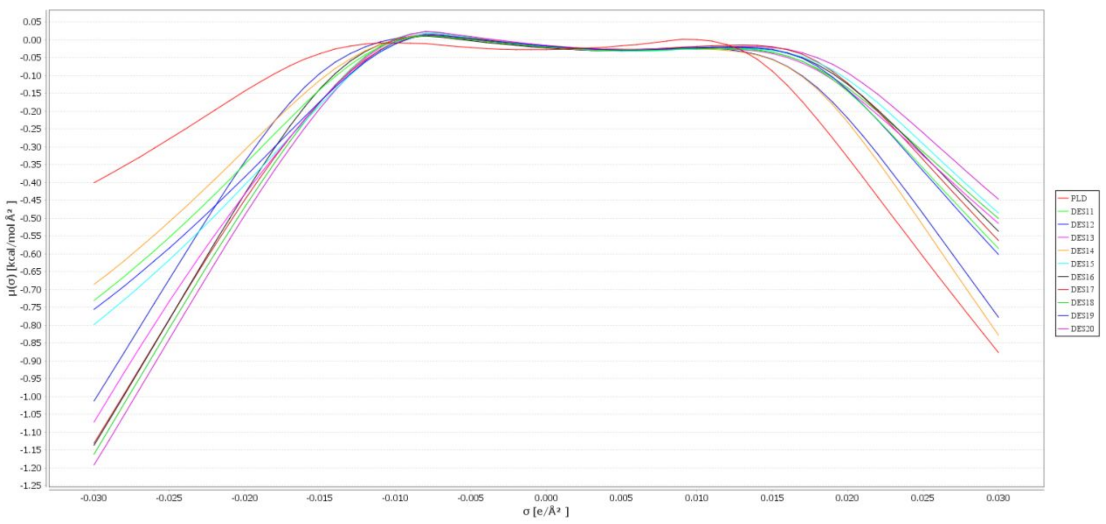

Figure S1 The σ-profile and σ-potential diagrams of deep eutectic solvents: (A) The σ-profile diagram of DES1~DES10; (B) The σ-potential diagram of DES1~DES10; (C) The σ-profile diagram of DES11~DES20; (D): The σ-potential diagram of DES11~DES20.

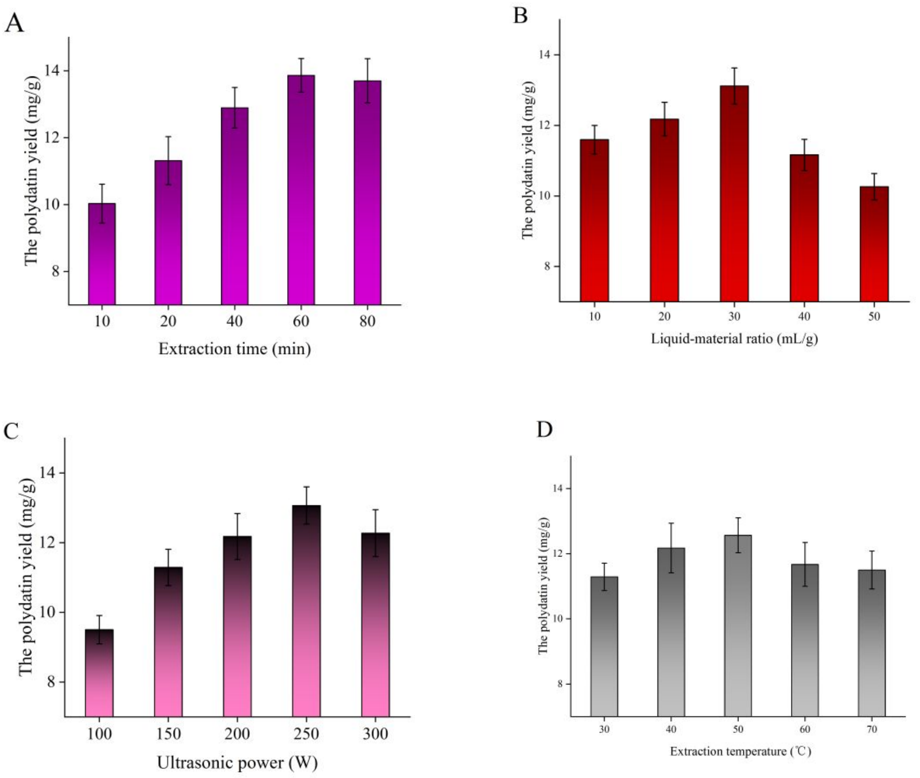

Figure S2. The results of single factor experiment. (A): Extraction time; (B): Liquid-material ratio; (C): Ultrasonic power; (D): Extraction temperature.

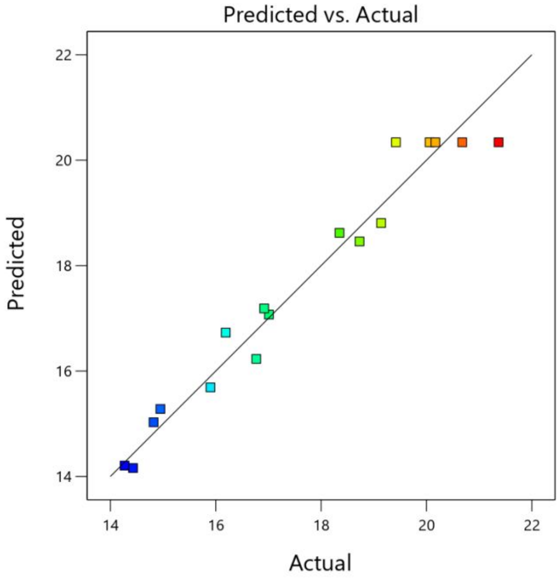

Figure S3. Predicted vs. actual plots

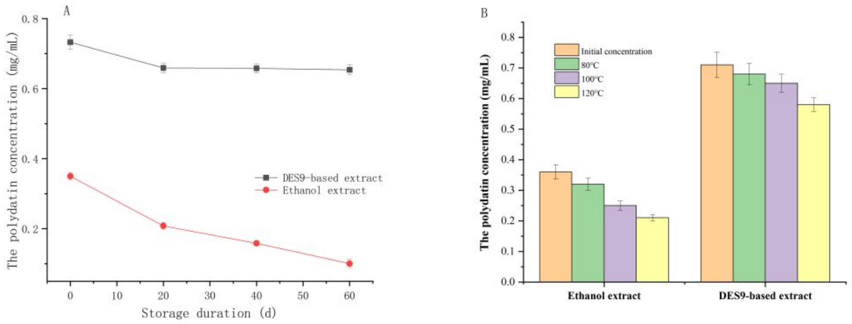

Figure S4. The stability of PLD in deep eutectic solvents and ethanol.

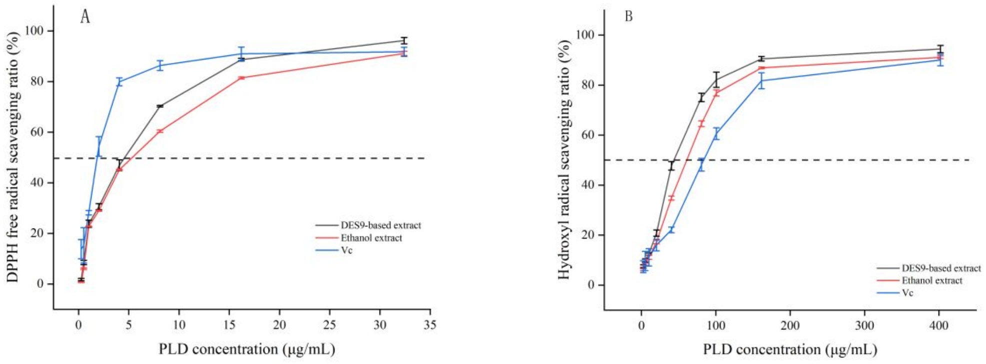

Figure S5. Results of antioxidant experiments. (A): The DPPH free radical scavenging ratio; (B) The hydroxyl radical scavenging ratio

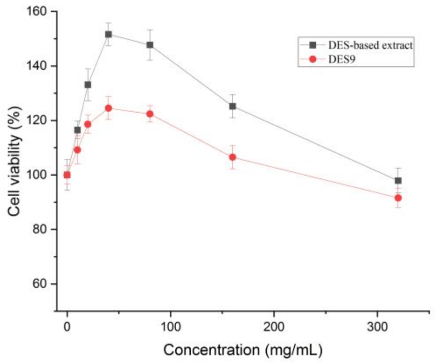

Figure S6. Cell viability of DES9-based extract and DES9 for HaCaT cells

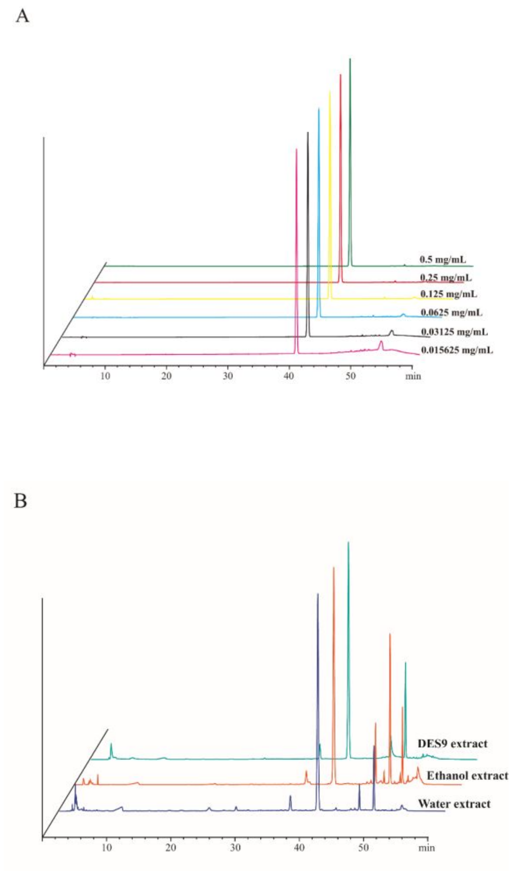

Figure S7. The HPLC diagrams. (A): Polydatin standard solutions; (B): Polydatin extracted by DES9, ethanol and water.

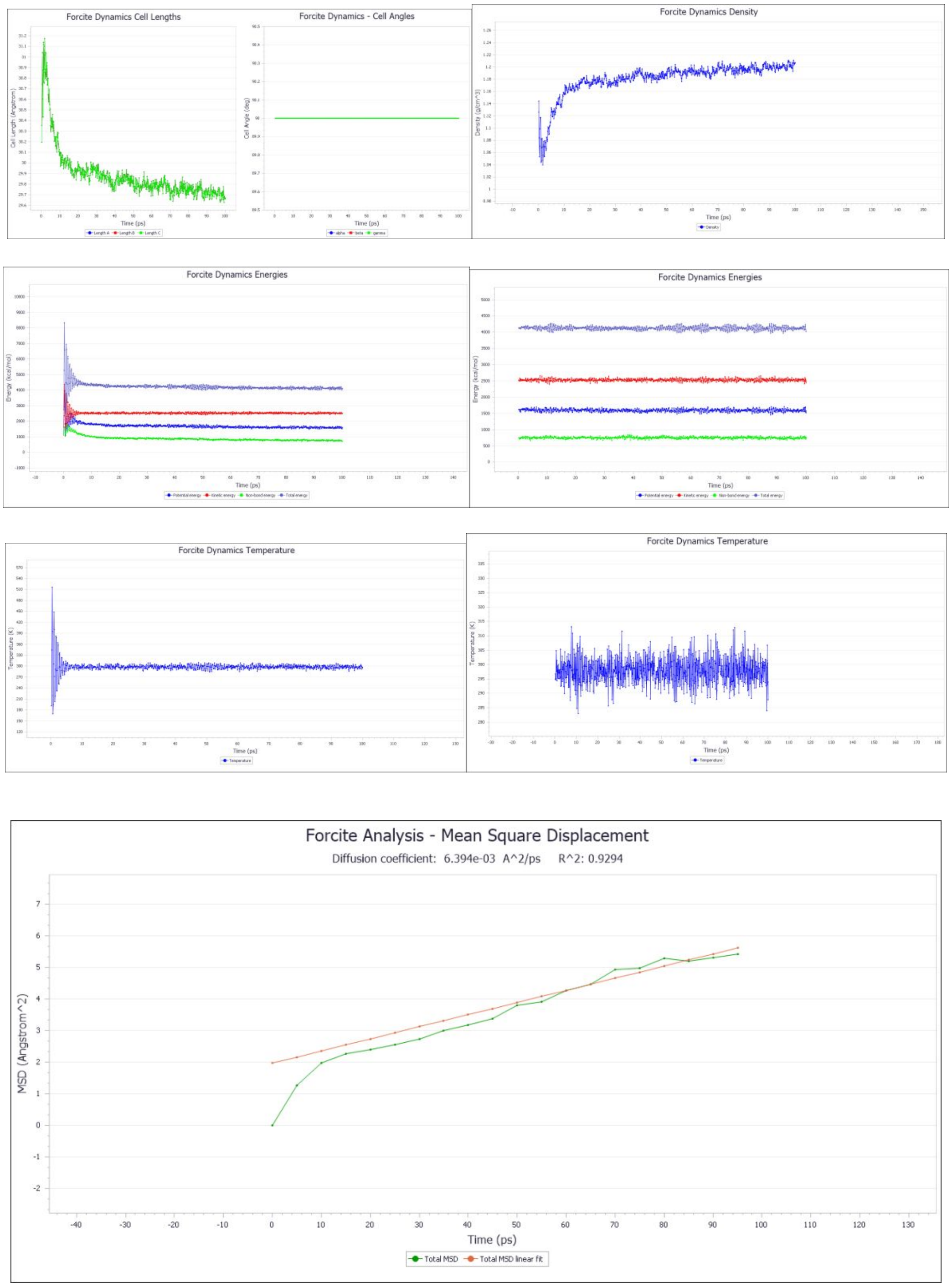

Figure S8 Molecular dynamics simulation of BET-Gly-PLD

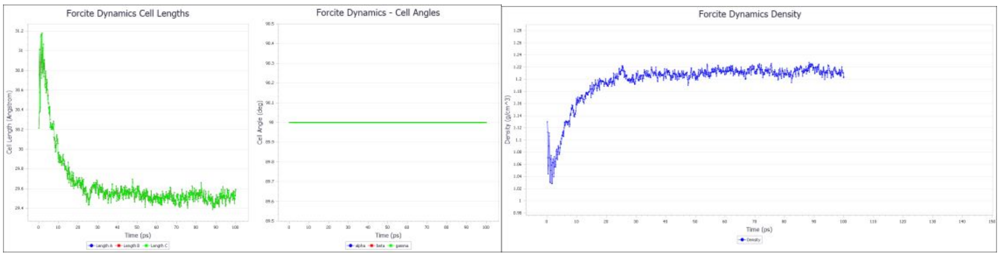

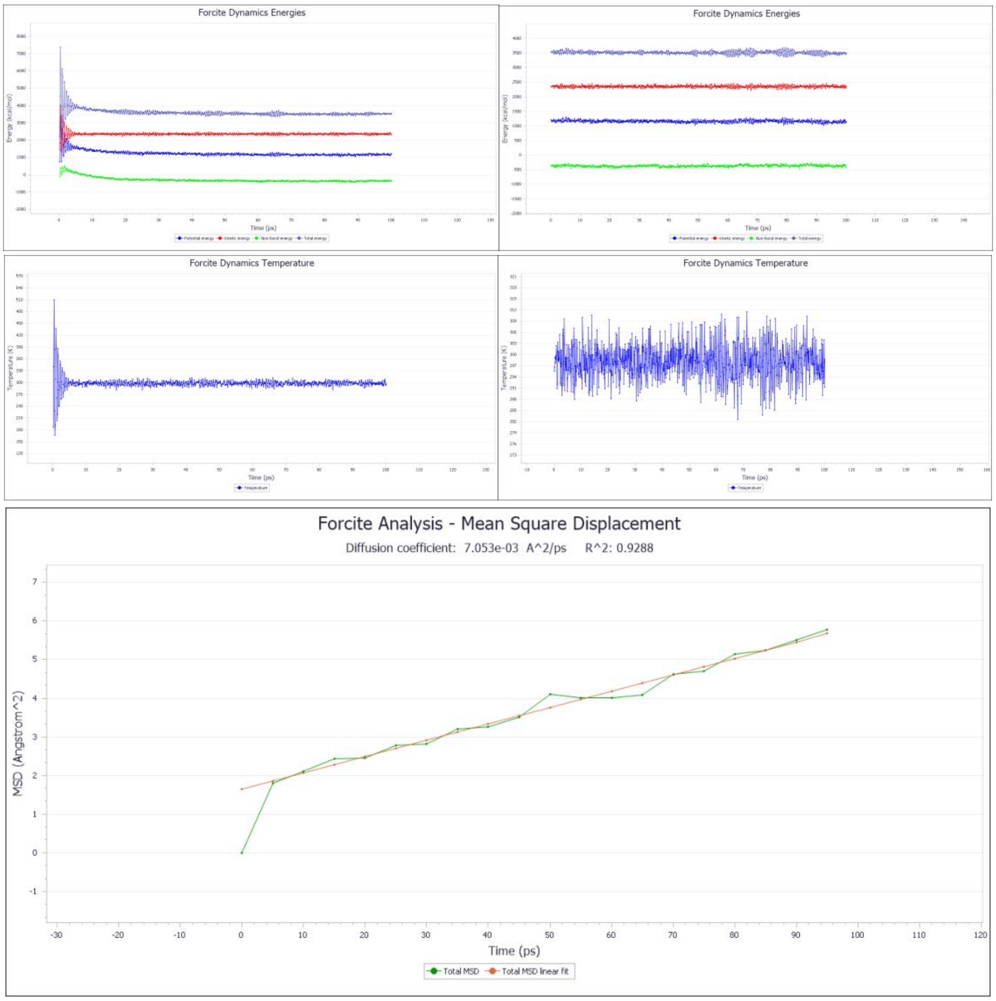

Figure S9 Molecular dynamics simulation of BET-LA-PLD

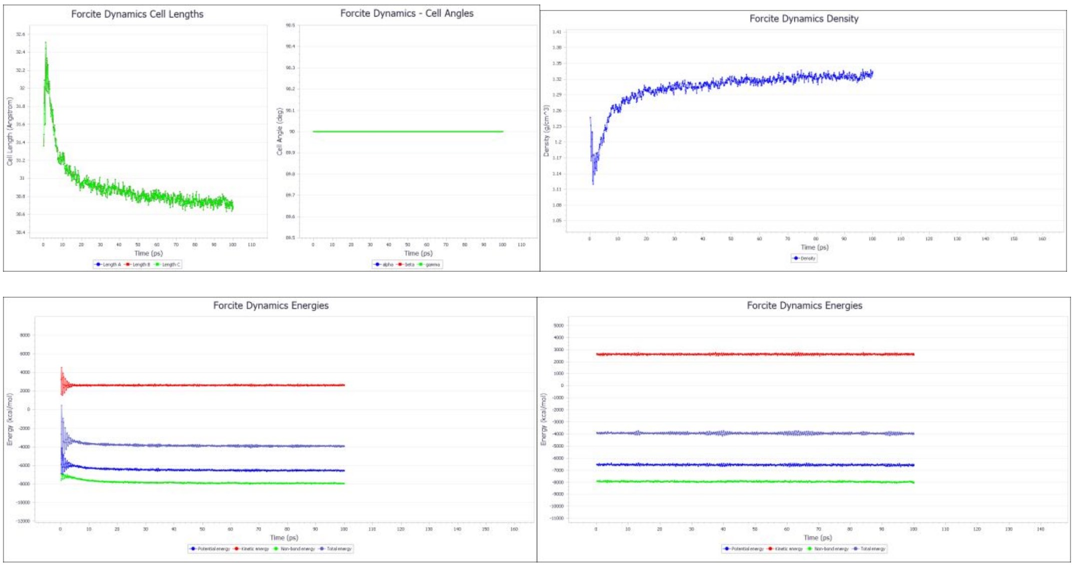

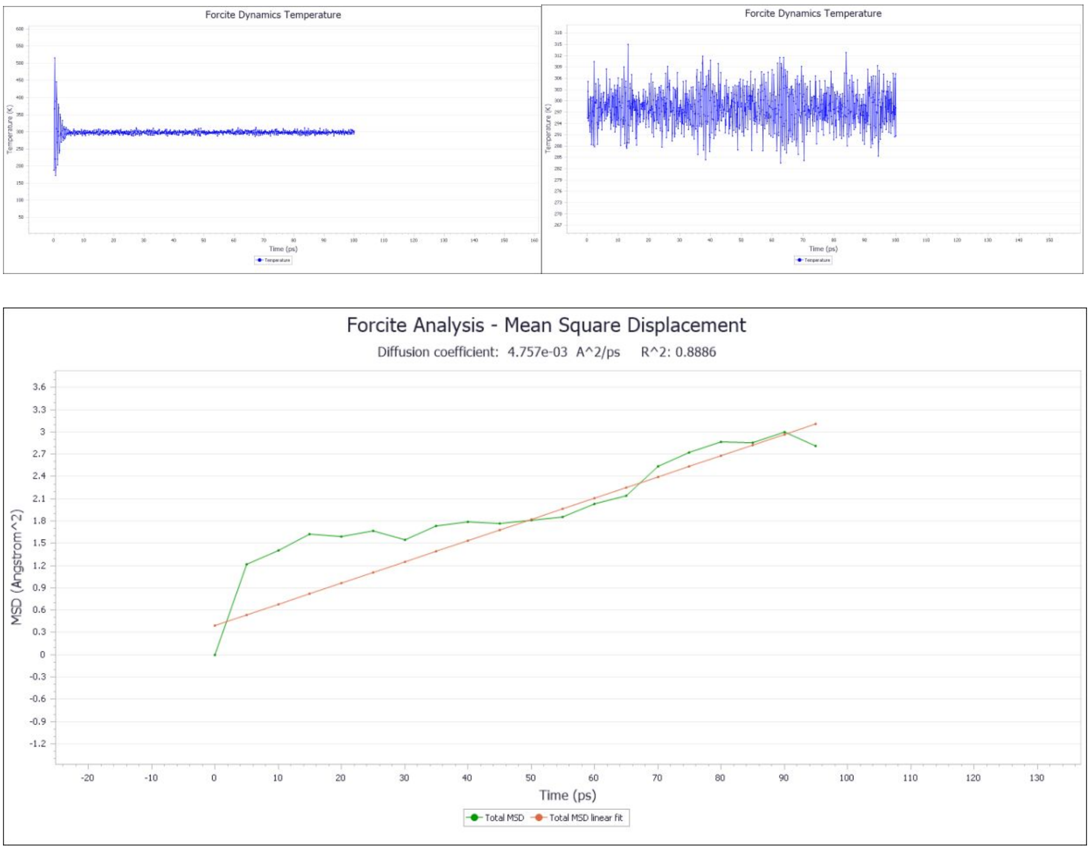

Figure S10 Molecular dynamics simulation of BET-MA-PLD

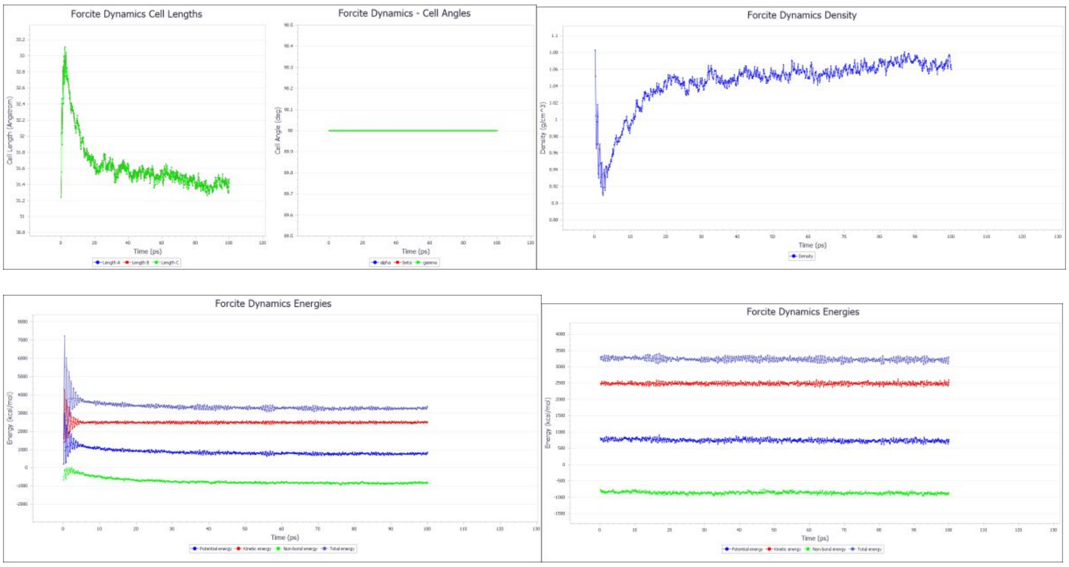

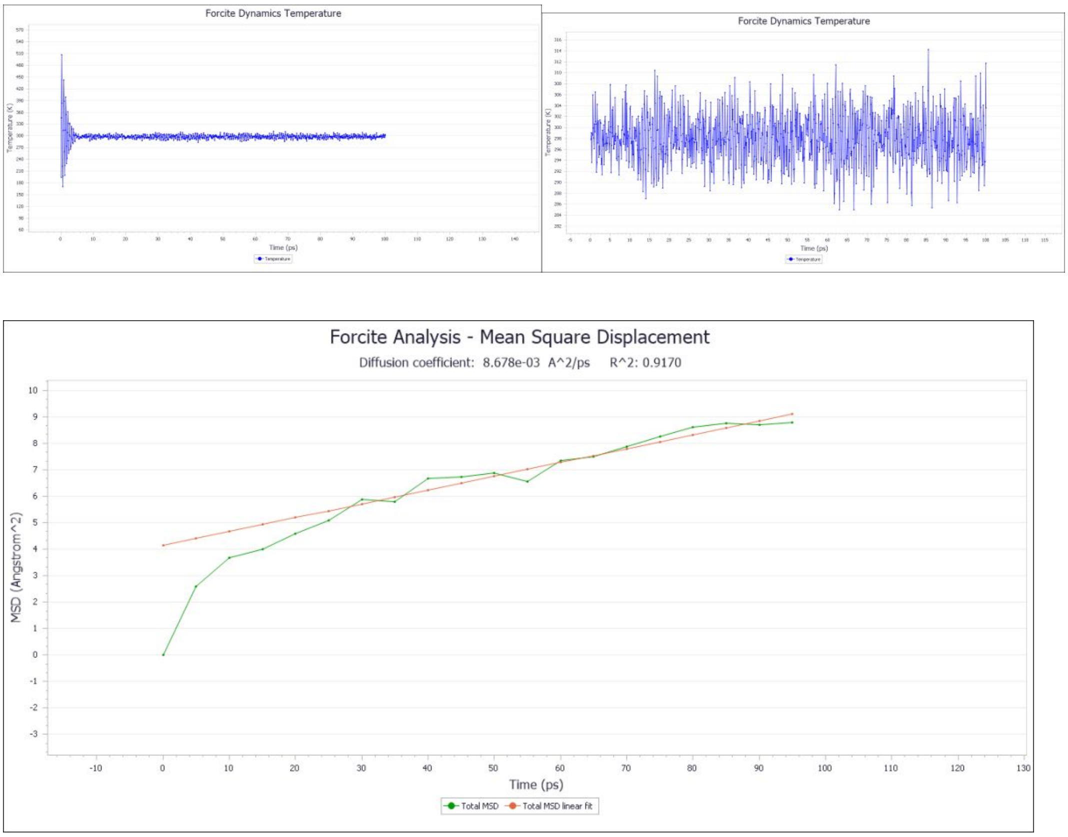

Figure S11 Molecular dynamics simulation of ChCl-LA-PLD

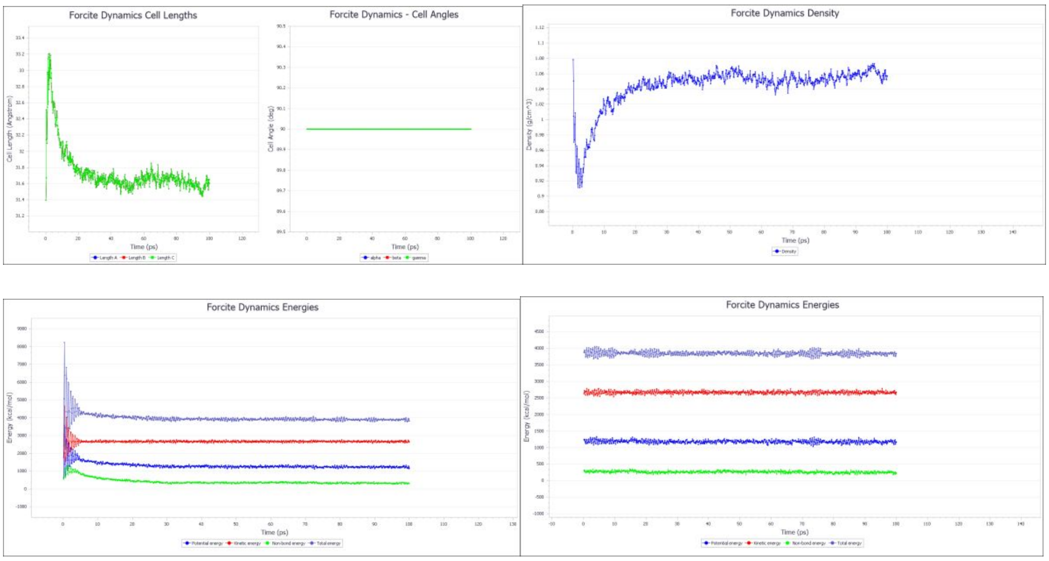

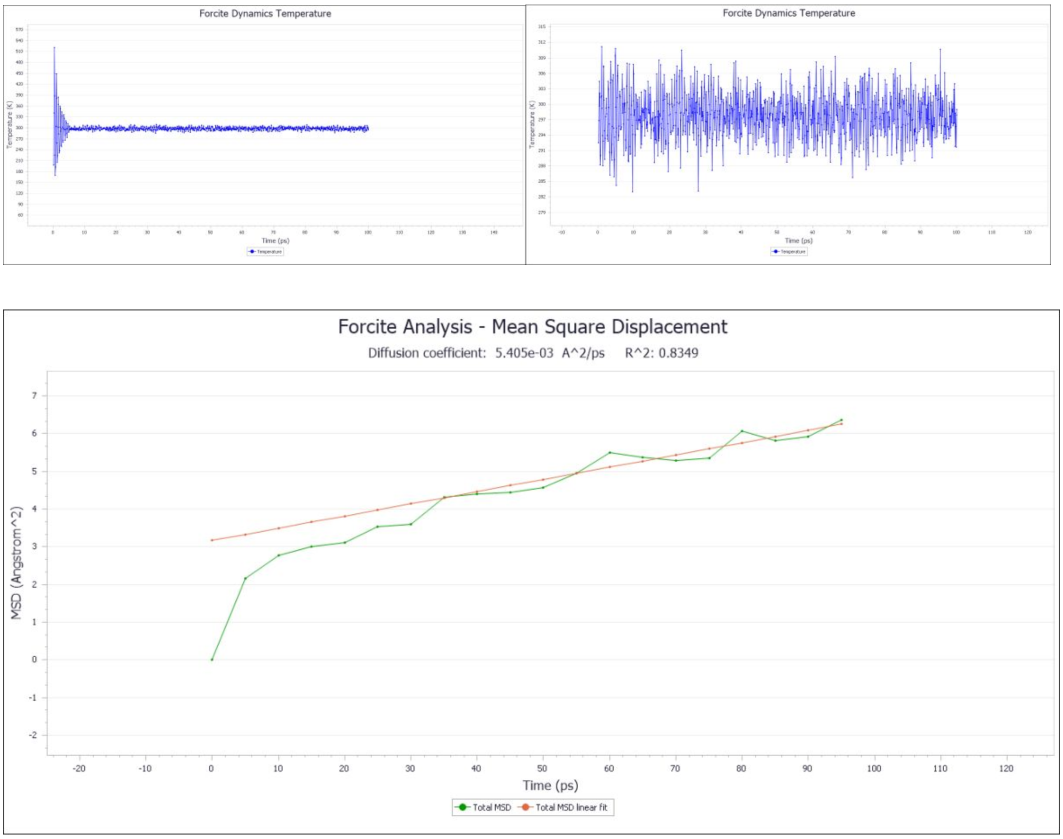

Figure S12 Molecular dynamics simulation of ChCl-Gly-PLD
